# A mathematical understanding of how cytoplasmic dynein walks on microtubules

**DOI:** 10.1098/rsos.171568

**Published:** 2018-08-08

**Authors:** L. Trott, M. Hafezparast, A. Madzvamuse

**Affiliations:** 1 Department of Mathematics, School of Mathematical and Physical Sciences, University of Sussex, Brighton BN1 9QH, UK; 2 School of Life Sciences, University of Sussex, Brighton BN1 9QH, UK; 3 School of Life Sciences, University of Sussex, Brighton BN1 9QG, UK

**Keywords:** motor protein, cytoplasmic dynein, microtubule, endocytosis, intracellular transport model

## Abstract

Cytoplasmic dynein 1 (hereafter referred to simply as dynein) is a dimeric motor protein that walks and transports intracellular cargos towards the minus end of microtubules. In this article, we formulate, based on physical principles, a mechanical model to describe the stepping behaviour of cytoplasmic dynein walking on microtubules from the cell membrane towards the nucleus. Unlike previous studies on physical models of this nature, we base our formulation on the whole structure of dynein to include the temporal dynamics of the individual subunits such as the cargo (for example, an endosome, vesicle or bead), two rings of six ATPase domains associated with diverse cellular activities (AAA+ rings) and the microtubule-binding domains which allow dynein to bind to microtubules. This mathematical framework allows us to examine experimental observations on dynein across a wide range of different species, as well as being able to make predictions on the temporal behaviour of the individual components of dynein not currently experimentally measured. Furthermore, we extend the model framework to include backward stepping, variable step size and dwelling. The power of our model is in its predictive nature; first it reflects recent experimental observations that dynein walks on microtubules using a weakly coordinated stepping pattern with predominantly not passing steps. Second, the model predicts that interhead coordination in the ATP cycle of cytoplasmic dynein is important in order to obtain the alternating stepping patterns and long run lengths seen in experiments.

## 1. Introduction

Cytoplasmic dynein 1 (hereafter referred to simply as dynein) is a protein complex which moves in the centripetal direction along microtubules, i.e. towards the minus ends of microtubules which are usually directed towards the cell centre, transporting cellular cargo such as vesicles and organelles, and is crucial for supporting events associated with cell division, cell survival and cell migration (see [1–5] for further details). Experimentally, it is known that during mitosis, dynein plays a key role in the positioning of spindles, focusing microtubules into poles, thereby regulating the spindle assembly check point. A large number of neurodegenerative diseases and developmental problems are now known to result from mutations in dynein or dynein-binding proteins [6–9]. Errors in the heavy chain of dynein, encoded by dynein cytoplasmic 1 heavy chain 1 (*DYNC1H1*) gene, have been implicated in spinal muscular atrophy with lower extremity predominance (SMA-LED), Charcot-Marie-Tooth disease type 2 (CMT2) and intellectual disability (reviewed in [8]; see also [9]). Investigations into mutations in dynein have shown particular behavioural differences, such as a decrease in velocity and distance travelled in a mouse strain known as ‘Legs at odd angles’ (*Loa*) [10–12]. Studies by Hafezparast *et al.* [10,13], have shown that the DYNC1H1^*F*580*Y*^ mutation in the *Loa* mouse strain negatively affects fast retrograde transport mediated by dynein, including an increase in pauses in motion. Work by Deng *et al.* [14], has shown that the *Loa* mutation gives rise to a lower affinity of dynein to dynactin, which regulates cargo binding and dynein processivity. The devastating effect of dynein malfunction presented in mutation studies on mouse models as well as in humans shows the need for greater understanding of the mechanics and processes used by dynein [8–10,13]. The dynein family is particularly interesting as it has evolved separately from other motor protein families, kinesin and myosin, and has a very different structure and mechanics (see [15] for a detailed review).

**Figure 1.**
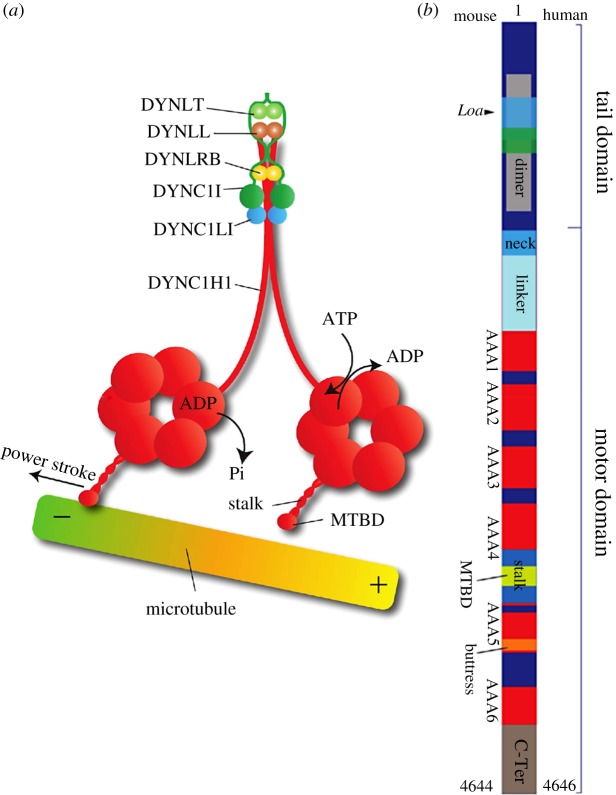
A schematic diagram of cytoplasmic dynein protein complex. (*a*) Cytoplasmic dynein is a protein complex consisting of two homodimerized heavy chains (DYNC1H1) and associated proteins intermediate (DYNC1I), light intermediate (DYNC1LI) and light chains (DYNLRB, DYNLL, DYNLT). The C-terminal portion of the heavy chain encompasses the microtubule binding (MTBD) and motor domains. The N-terminal domain is responsible for the heavy chain homodimerization and binding of accessory proteins to the complex. (*b*) DYNC1H1 domains and the site of the Legs at odd angles (*Loa*) mutation in the mouse protein.

The largest components of the dynein complex are two homodimerised heavy chains, each of which is made up of a tail and a motor domain. The N-terminal tail domain (residues 1 to approx. 1400) binds to other regulatory and structural components of dynein, through which cargo and adaptor proteins bind to the complex (figure 1). The structure of the head comprises a linker, a ring of six ATPase domains associated with diverse cellular activities (AAA+), from which a microtubule-interacting stalk region and a buttress extend, and a C-terminal sequence [16]. The linker is located between the tail and the ring and spans across the top face of the ring before bending down the side of the AAA1 domain of the ring. It plays a key role in the nucleotide-dependent power stroke of the motor by switching from bent to straight conformations [7,17]. Only four of the AAA+ domains of the motor domain are thought to bind ATP [8,18–22]. This is in contrast to kinesin and myosin, each of which have a single ATPase-binding site per motor domain [23]. The coupling of ATP hydrolysis and force generation is not yet fully understood, although recent progress has been made with structural cycles being suggested by Carter [16] and Lin *et al.* [24] as well as by Nicholas *et al.* [25] and DeWitt *et al.* [26] on the role of the AAA3 domain (see [3,27] for detailed reviews on dynein's mechanism). The stalk is formed of an anti-parallel-coiled coil, which extends from between the AAA4 and AAA5 domains ending with a microtubule binding domain (MTBD); the recently identified component labelled a *strut* or *buttress* is proposed to support the stalk under load [8,18,19]. The stalk-coiled coil acts as a communication pathway between the AAA rings and the MTBDs.

It must be noted that dynein-driven transport of cargos along microtubules requires other components such as the cofactor dynactin and other regulatory proteins [1,2,28]. For example, recent experimental observations show how dynactin recruits two dimeric dyneins for faster movement, supporting the notion that dynein stepping patterns on microtubules could be influenced by such cofactors [28,29]. The emerging evidence on the structure and function of dynein-dynactin are providing growing insight into how these two act together to carry cargos [2,28–33]. In this study, we will not take into account other complex processes associated with dynein structure and function and these include the role of cofactors in the dynein transport mechanism, dynein auto-inhibition and activation (by phi-particle, for example), etc. [34,35]. Instead, we will focus on the whole dynein structure and how it walks on microtubules. The mathematical framework presented allows for other cofactors or processes to be included in future studies; however, such studies are beyond the scope of this study.

Hence, in this paper we derive a general integrative mechanistic model for dynein that describes the qualitative and quantitative results observed in experiments and could be applied to particular dynein complexes through parameter variations or functions. The form of stepping pattern used by dynein and the possibility of interhead coordination is modelled and discussed. Hence, this article is structured as follows. In the next section, we review experimental observations setting premises for the derivation of the integrative mechanical model based on physical principles. Section 3 reviews briefly current mathematical models for dynein transport from the cell membrane towards the nucleus. It is here that we contrast our model with those in the literature. The main thrust of our work is presented in §4 where we formulate from first principles the physical mechanical model integrating the temporal dynamics of the individual components that include the cargo, tail domain, AAA+ rings and MTBDs. For simplicity, we formulate our modelling on a one-dimensional microtubule, leaving extensions to multi-dimensions for future studies. Similarly, modelling of multiple dyneins walking on microtubules [7] is omitted and forms part of our future studies. In §5, stochasticity is introduced into the model to account for the random binding of ATP to either of the two motor domains, and numerical simulations for the model equations are presented. Within this section, qualitative and quantitative agreements with some experimental observations are discussed, and the effect of interhead coordination is explored. Backward stepping, variable step size and dwelling are further modelled, and numerical simulations exhibit this stepping behaviour. Furthermore, we make predictions amenable for experimental manipulations. Finally, in §6, we discuss the implications of our modelling to understanding mechanisms for dynein-mediated transport.

## 2. Experimental observations

Experimental studies (using total internal reflection fluorescence, X-ray crystallography and high-resolution cryo-electron microscopy) on how cytoplasmic dynein motors move along microtubules transporting cargo to the nucleus can be subdivided into two parts. Those that focus on the single molecule motility properties of dynein [3,21,36,37] and those that focus on the mechanical structural dynamics of dynein [2,20,21,35,38]. Furthermore, experimental data both *in vivo* [39–41] and *in vitro* [2,4,42] are performed and obtained on different species (e.g. yeast [3,35], *Dictyostelium discoideum* [2,4,7,20,23], human [38,42], *Saccharomyces cerevisiae* [21,36,37], etc.). Moreover, studies are carried out either in one-dimension [37] or two-dimensions [4,36,37]. In the work by Qiu *et al.* [37], two-dimensional particle tracking shows dynein's two motor domains can step both alternatively and non-alternatively in time and either passing or not passing in space. One-dimensional tracking results are then extrapolated from the two-dimensional data through a projection operator in the direction of motion along the microtubules axis. Given these different experimental conditions, it is therefore a significant challenge to come up with a single mathematical model that can capture all these processes. Given that the single molecule motility dynamics are part of the whole dynein structure, we therefore propose to study the whole dynein structure with the rationale that different experimental conditions can either be modelled through parameter variations and functions or through appropriate extensions of the model to take into account other processes associated with dynein transport that are not the subject of our study. Having this in mind, we therefore present some of the most recent experimental observations of cytoplasmic dynein with an eye to making comparisons with the model where appropriate. Moreover, the structural mechanism of cytoplasmic dynein's processive stepping along MTs is unclear and sets the motivation for this study.

Recent experimental studies on the structure of yeast and *Dictyostelium discoideum* dyneins include works by Bhabha *et al.* [35], Carter *et al.* [18] and Schmidt *et al.* [21], which allowed for detailed visualization of the AAA domain and linker movements. On the other hand, studies on yeast and *Dictyostelium discoideum* dyneins show that the replacement of the tail with glutathione S-transerase yields a simpler dimer that still processively steps along MT [7]. It is known that perhaps the most striking feature of stepping dynein is the huge flexibility between the ATPase domain and the track-binding domain, which is in contrast to kinesin and myosin motors [7]. In order to provide quantitative data on dynein's processive stepping along MTs, Dewitt *et al.* [36] and Qiu *et al.* [37] tracked fluorescent-tagged yeast cytoplasmic dynein in two-dimensions, and their studies suggested uncoordinated stepping pattern by the two heads but that they also must communicate (i.e. coordinate) as the properties of dimerization to MTs are different from those of monomers [7]. Statistically, their studies found that 74% of dynein steps were taken by each of the two heads alternating in time (i.e. the motor domains' relative temporal behaviour) and that 83% did not pass each other (in terms of their relative spatial behaviour), suggesting that dynein may move predominantly by passing rather than in an alternating fashion [37]. This is in contrast to the processive kinesin and myosins which walk hand-over-hand [4,43–46]. Both papers also found that the leading head was more likely to be to the right of the lagging head along the direction of movement [36,37]. Furthermore, experimental observations show that dynein has a variable step size, with the majority of steps being 8 nm in distance [36,37,47–49]. It must be noted, however, that this reflects the position of the tail of dynein rather than the motor domain, and further investigations have shown that the motors move with a usual step size of around 16 nm [36,37,48]. Moreover, we note that dynein steps are not always parallel to the microtubule and usually have off-axis components [10,36,37,48], and dynein can also take backward steps [47,48]. Carter *et al.* [50] proposed that the stalk acts as a tether in the stepping process and that the MTBD determines the direction of the step, while Redwine *et al.* [51] proposed that conformational changes in the MTBD lead to movement in the linker domain and hence displacement of the MTBD.

We note that the experimental studies by DeWitt *et al.* [36] and Qiu *et al.* [37] revealed the stepping behaviour of *yeast* cytoplasmic dynein; however, throughout this paper, we will also consider mammalian cytoplasmic dynein; hence, the model is not restricted to specific species. Furthermore, due to the fact that at the stalk–stalkhead junction, the hinge is located close to the MT surface (two-dimensional geometry), the dynein head swings over a wide range of approximately 20 nm compared with approximately 8 nm spacing between the binding sites on the MT. Studies by Imai *et al.* [7] suggest that experiments such as those by DeWitt *et al.* and Qiu *et al.*, where fluorescent tags are attached to the heads for stepping studies, may not reliably report the position of the MT-bound stalkheads. During the processive stepping, the flexibility between the ATPase and track-binding domains may allow for the stalkhead to detach from its partner motor with greater freedom to explore the MT surface for locating its next binding site. Hence, these studies provide a structural basis for a wide range of step sizes (variable) seen in dynein stepping studies [7]. As a proof of concept, we will nevertheless compare our results to those of DeWitt *et al.* and Qiu *et al.* (with the caveat above and noting also that our model is formulated in one dimension) as our theoretical study is a first stepping stone in modelling the integrated dynein structure. The framework can easily be applied to specific dynein stepping studies when quantitative experimental data are available. For example, by including a linker into the modelling, results could be compared quantitatively with those obtained in studies by Cleary *et al.* [4].

While these experimental studies enable detailed understanding of dynein's structure [2,4,7,18–21,35] and transport mechanism [3,34,36,37,42,47,48,52]; to our knowledge, few models have been developed to describe such observations [53–55]. Our results bridge this gap, by presenting a robust integrative mechanical and stochastic model describing the stepping behaviour of cytoplasmic dynein.

## 3. Overview of current mathematical models describing transport processes for cytoplasmic dynein

Recently, there has been a notable increase in mathematical models studying endocytosis and cytoplasmic dynein and these include models proposed by Ashwin *et al.* [56], Smith & Simmons [57], Šarlah & Vilfan [54], Mukherji [58] and Tsygankov *et al.* [55,59]. For example, the Smith and Simmons model [57] allows for motion of dynein along the microtubules when cell organelles and vesicles, referred to as particles, are attached and freely diffuse when they are not. Under this framework, they consider particle densities in one dimension, described by reaction-diffusion-transport equations. This model helps us to understand the macroscopic behaviour of endosomes and not the particular mechanisms of dynein. In the model proposed by Ashwin *et al.* [56], they consider a single microtubule for which the motor protein dynein moves to the minus end (i.e. towards the nucleus) when bound and is carried by the motor protein kinesin to the plus end (towards the cell periphery). They assume that right and left moving motors pass without interaction, but there is an *exclusion principle* enforcing that a motor can only move forward if the site ahead is free of motors of the same type. They discretize the microtubule into two tracks and use a mean field approximation and further simplifications. This model describes the behaviour of a population of dynein; it does not consider a more detailed model involving a single dynein within the transport process and therefore is not able to quantify the temporal behaviour of the individual components of the dynein structure.

Single dynein models have been considered by Mukherji [58], Tsygankov *et al.* [55,59] and Šarlah & Vilfan [54]. Mukherji [58] and Tsygankov *et al.* [59] study the mechanochemical cycle of dynein which is essential for understanding dynein's behaviour. An extension to the model by Tsygankov looks at the bending energies of dynein using Langevin equations and couples this to the biochemical reactions modelled previously [55,59]. Šarlah and Vilfan propose a winch model for cytoplasmic dynein which couples an elastomechanical model to a kinetic model of the ATPase cycle. For the elastomechanical model, they consider elastic energies within the complex, interaction between the two motors and work done against external load. Monte Carlo methods are then used to find the shapes of the complex with minimum energy. In this work, we propose an alternative framework; our aim is to derive a model that studies dynein's progress along the microtubule over time as opposed to mean run lengths and velocities, taking a mechanical approach with the long-term aim of modelling the mechanical effects of mutations on dynein. A similar approach has been studied for kinesin by Hendricks *et al.* [60] and by us in a previous work by Crossley *et al.* [53]. We will study the whole structure, looking at the positions of the cargo carried by dynein, the tail domain, AAA+ rings and MTBDs comparing our results to data from different experiments tracking single components of the transport process. We will also consider dynein in general, allowing the model to be applied later to particular dynein species through the use of parameter variations. Unlike this current work, the previous study was devoid of any statistical analysis which forms the bulk of the current modelling approach. The main contributions of this study are the stochastic multiscale modelling, as opposed to the use of continuous functions to model binding and ATP force previously studied, and the introduction of the tail component. Furthermore, we model for the first time variable stepping, including heads being able to move independently, not in a strictly coordinated pattern. Other processes such as variable steps, backward stepping and dwelling times are also modelled for the first time.

## 4. Derivation of the mechanical model

**Figure 2.**
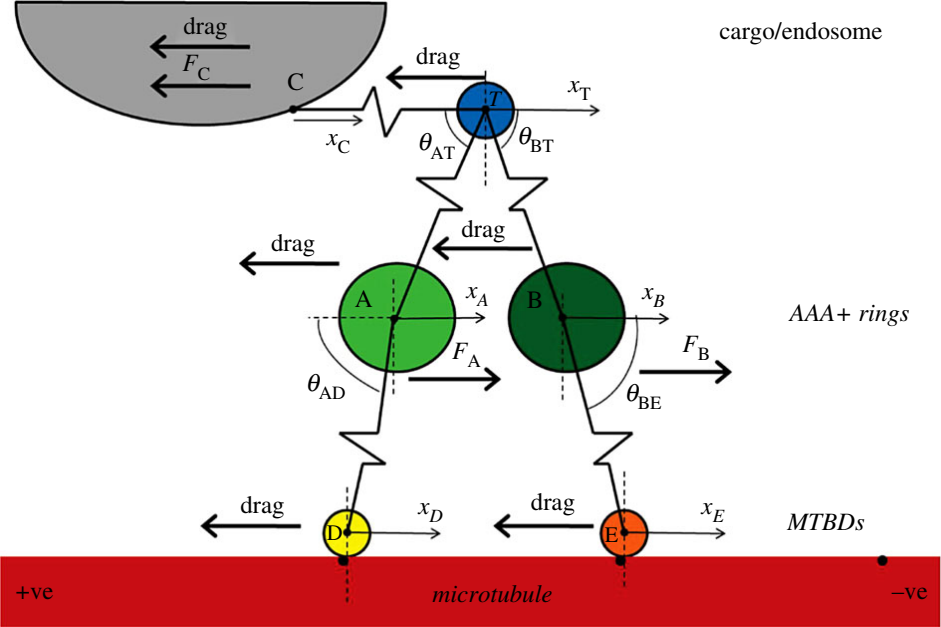
A schematic diagram of the mechanical model (adapted from [53]). The cargo is modelled as a sphere (grey) and regulators of binding to dynein are modelled as part of this cargo. The binding of the cargo to the tail domain is modelled by a spring. The tail of dynein is modelled by a sphere (blue) connected by two springs to the AAA+ rings. The AAA+ rings, depicted in green, and the MTBDs, depicted in yellow and orange, are modelled as spheres. The stalks are modelled as springs. The microtubule is modelled as a line (red).

Following our previous study by Crossley *et al.* [53], we derive from first principles a system of six second-order nonlinear ordinary differential equations (ODEs) to model the transport mechanisms of a single dynein acting on a cargo. Let *x*_C_(*t*), *x*_T_(*t*), *x*_*A*_(*t*), *x*_*B*_(*t*), *x*_*D*_(*t*) and *x*_*E*_(*t*) denote the positions of the cargo, tail, AAA+ rings A and B, and the MTBDs D and E, respectively, at time *t* ∈ [0, *T*_Final_] for some end time *T*_Final_ > 0. We note that throughout this work, time is measured in nanoseconds and length in nanometres. For simplicity, we omit the units when stating the time and length variables. The coordinates *x*_*A*_ and *x*_*D*_ represent one head domain of dynein with the coordinates *x*_*B*_ and *x*_*E*_ representing the other head (figure 2). We model the microtubule as a one-dimensional line with binding sites 8 nm apart; we only consider motion along this line. We make the following assumptions:

— The mass of the cargo remains constant and is modelled as a sphere with small Reynolds number. This is a significant assumption for experiments *in vivo*; however, it is applicable to *in vitro* experiments with beads.
— Any regulators of cargo binding, such as dynactin, are modelled as part of the cargo.
— The tail domain is modelled as two identical springs, from the AAA+ rings, connected to a sphere with small Reynolds number and constant mass. The linker is modelled as part of these springs. The binding between the tail and the cargo is modelled via another spring connecting the tail domain to the cargo.
— The AAA+ rings are identical and modelled as spheres with small Reynolds number whose masses remain constant.
— The stalks are modelled as two identical springs. We model the strut or buttress as part of this spring.
— The MTBDs are identical and modelled as spheres with small Reynolds number whose masses remain constant.

See figure 2 for a schematic diagram illustrating the whole structure on which the mathematical model is based and table 1 for a list of parameter values. We make four simplifying assumptions that will be relaxed in future studies (see remark 4.1):

**Table 1.** Dimensional parameters and the primary values used in the mathematical model. The drag coefficients are given by *γ*_*i*_ = (6*πηR*_*i*_) MDa/ns for *i* = *C*, *T*, *M*, *S* with *η* and *R*_*i*_ given below. The binding sites are described by *p*_*k*+1_ = (*p*_*k*_ + 8) nm where *p*_0_ = (*L*_C_ + *L*_T_ − 4) nm, with *L*_C_, *L*_T_ given below.

parameter	description	value	ref.
*M*_C_	mass of the cargo	2 MDa	estimated
*M*_T_	mass of the tail component	0.14 MDa	estimated
*M*_*M*_	mass of the AAA+ ring	0.5 MDa	estimated
*M*_*S*_	mass of the MTBD	0.03 MDa	estimated
*R*_C_	radius of the cargo	460 nm	[47]
*R*_T_	radius of the tail domain	3 nm	[61]
*R*_*M*_	radius of the AAA+ ring	6.5 nm	[61,62]
*R*_*S*_	radius of the MTBD	1.5 nm	[62]
*L*_C_	unstressed length between the cargo and tail	12 nm	[61]
*L*_T_	unstressed length between the AAA+ ring and tail	8 nm	[61]
*L*_*S*_	unstressed length between the AAA+ ring and MTBD	15 nm	[61]
*K*_C_	spring constant between the cargo and the tail	1 MDa ns^−2^ (≈1.66 pN nm^−1^)	estimated
*K*_T_	spring constant between the tail and the AAA+ ring	1 MDa ns^−2^ (≈1.66 pN nm^−1^)	estimated
*K*_*S*_	spring constant between the AAA+ ring and the MTBD	10 MDa ns^−2^ (≈16.61 pN nm^−1^)	estimated
*F*_C_	external force exerted on the cargo	0 MDa nm ns^−2^ (=0 pN)	assumption
*η*	viscosity of the cytoplasm	1.2 MDa nm^−1^ ns (≈1.99 cP)	[63]
*L*_ATP_	unstressed length between the binding sites	16 nm^a^	estimated [19,61]
*K*_ATP_	ATP unbound state spring constant	10 MDa ns^−2^ (≈16.61 pN nm^−1^)^b^	estimated
*γ*_ATP_	ATP unbound state drag coefficient	10 MDa ns^−1^ (≈16.61 pN ns nm^−1^)^b^	estimated
*θ*_AD_	angle of the stalk between AAA+ ring A and MTBD D	53°	[61]
*θ*_BE_	angle of the stalk between AAA+ ring B and MTBD E	53°	[61]
*θ*_AT_	angle of the spring between AAA+ ring A and the tail domain	33°	[61]
*θ*_BT_	angle of the spring between AAA+ ring B and the tail domain	33°	[61]

^a^In §5.4, we use *L*_ATP_ = 8 nm to allow for step sizes in multiples of 8 nm.

^b^Note that in §5.3 we explore a range of values for *K*_ATP_, 10–1000 MDa ns^−2^ and *γ*_ATP_, between 1 and 1000 MDa ns^−1^. Some of the values such as the ATP unbound state spring and drag coefficients *K*_ATP_ and *γ*_ATP_, respectively, are estimated by trial-and-error method.

— The spring between the cargo and tail domain is parallel to the microtubule.
— The springs between the tail domain and AAA+ rings are at a fixed angle to the microtubule.
— The stalks are at a fixed angle to the microtubule.
— There are no external forces acting on the cargo from other motor proteins nor an optical trap.

**Remark 4.1.**

The simplifying assumption of fixed angles means that the AAA+ rings and cargo will move according to the extension and relaxation of the springs horizontally. This is an appropriate assumption for the model while we remain in one space dimension but will need to be considered when moving to higher dimensions. It is likely that there is some rigidity within the complex with regard to these angles, with the main variation arising from the conformational change under ATP hydrolysis. The model has been solved in two space dimensions with variable angles, but gives similar results to the simpler model presented here (see the electronic supplementary material for further details).

It must be noted that structural studies show that both stalks are tilted towards the plus-end and more or less in parallel orientation to each other (rather than pointing towards each other as depicted in figure 2). The mathematical framework can be modified easily to take into account this particular structure with no changes in the numerical results and model predictions (see the electronic supplementary material for further details on the model reformulation and the corresponding numerical results). Hence, our studies confirm similar results if stalks are assumed to be tilted towards the plus-end with angles ranging between 41.9° ± 13.7° [2,7,29,34,64,65]. In order to account for the angles of the dynein's off-axis steps, it is necessary to consider a two-dimensional model. The two-dimensional model will allow us to investigate whether dynein has a preferential stepping behaviour, either right or left on the microtubule surface. Such an analysis is not possible within the one-dimensional set-up proposed in this study.

To proceed, using Newton's Second Law we study the net forces acting on the system. For the cargo, there is a spring force, viscous drag and an external force acting on it. By Hooke's Law, we take the spring force to be:

4.1 $$F_{\mathrm{Spring}}(t)=K_{C}(x_{T}(t)-x_{C}(t)-L_{C}),$$

where *K*_C_ is the spring constant and *L*_C_ is the natural length. We obtain the viscous drag by Stokes' Law:

4.2 $$F_{\mathrm{Drag}}(t)=\gamma_{C}\frac{dx_{C}}{dt},$$

where the damping coefficient *γ*_C_ = 6*πηR*_C_ with *η* the viscosity and *R*_C_ the radius of the cargo. For completeness, we include an external force *F*_C_ that is exerted on the cargo, although throughout the model this is assumed to equal zero. Therefore, the equation of motion for the cargo can be modelled by

4.3 $$m_{C}\frac{d^{2}x_{C}}{dt^{2}}=K_{C}(x_{T}-x_{C}-L_{C})-F_{C}-\gamma_{C}\frac{dx_{C}}{dt}.$$

The equations of motion for the tail domain and AAA+ rings can be derived similarly. Therefore, we obtain the following system of ordinary differential equations for the cargo, tail and AAA+ rings, respectively:

4.4 $$\;m_{C}\frac{d^{2}x_{C}}{dt^{2}}=K_{C}(x_{T}-x_{C}-L_{C})-F_{C}-\gamma_{C}\frac{dx_{C}}{dt},$$

4.5 $$\;m_{T}\frac{d^{2}x_{T}}{dt^{2}}=K_{T}(x_{B}-x_{T}-L_{T}\cos(\theta_{\mathrm{BT}}))-K_{T}(x_{T}-x_{A}-L_{T}\cos(\theta_{\mathrm{AT}}))-K_{C}(x_{T}-x_{C}-L_{C})-\gamma_{T}\frac{dx_{T}}{dt},$$

4.6 $$\;m_{M}\frac{d^{2}x_{A}}{dt^{2}}=K_{T}(x_{T}-x_{A}-L_{T}\cos(\theta_{\mathrm{AT}}))-K_{S}(x_{A}-x_{D}-L_{S}\cos(\theta_{\mathrm{AD}}))-\gamma_{M}\frac{dx_{A}}{dt}$$

4.7 $$\mathrm{and}\;m_{M}\frac{d^{2}x_{B}}{dt^{2}}=K_{S}(x_{E}-x_{B}-L_{S}\cos(\theta_{\mathrm{BE}}))-K_{T}(x_{B}-x_{T}-L_{T}\cos(\theta_{\mathrm{BT}}))-\gamma_{M}\frac{dx_{B}}{dt}.$$

We wish to model the mechanics of ATP hydrolysis on the motor domain of dynein. The binding of ATP occurs randomly and is followed by microtubule release of the corresponding MTBD and a recovery stroke towards the next binding site [16,24]. Hence, we will assume that there are two MTBD states:

— Bound: This is defined to be when the MTBD is bound to the microtubule and hence is stationary.
— Unbound: Defined to be when the MTBD is unbound from the microtubule and undergoing the recovery stroke towards the next binding site.

**Figure 3.**
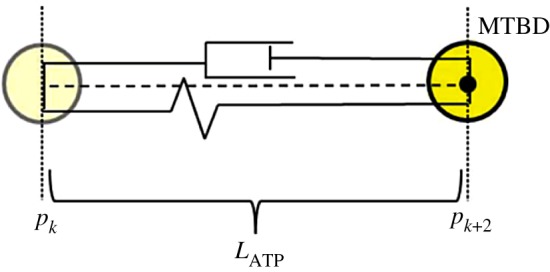
A schematic diagram of the dashpot-spring model for the conformational change in dynein resulting from the binding of ATP. For the time interval [*t*_*i*_, *t*_*i*+1_], the MTBD is at the binding site *p*_*k*_ at time *t*_*i*_ and moves to the binding site *p*_*k*+2_ by time *t*_*i*+1_ with a step size of *L*_ATP_.

It is hypothesized that ATP hydrolysis induces a conformational change in dynein, potentially causing a 37° kink in the stalk [19]. Hence, for the unbound state the conformational change is modelled by a dashpot and spring acting solely on the MTBD (figure 3) [66]. It is assumed that this force is independent of the particular interval on the microtubule, defined by *x* ∈ [*p*_*k*_, *p*_*k*+2_], and is identical for the two head domains. Binding sites are taken to be *p*_2*k*_ for MTBD D and *p*_2*k*+1_ for MTBD E with *k* = 0, 1, 2, … and *p*_2*k*+1_ − *p*_2*k*_ = 8 nm, binding sites are 8 nm apart on the microtubule with each MTBD binding to distinct binding sites that are 16 nm apart. The current model is one dimensional and hence it is assumed that this force acts only in the horizontal direction. The force produced by the dashpot is proportional to the velocity and the spring force is proportional to the displacement, hence

4.8 $$F_{\mathrm{ATP}}(x(t))=-\gamma_{\mathrm{ATP}}\frac{dx}{dt}+K_{\mathrm{ATP}}(L_{\mathrm{ATP}}-(x(t)-x(0))),$$

where *γ*_ATP_ and *K*_ATP_ are parameters determining the size of the ATP force, with estimated values given in table 1. These parameters are estimated by trial and error. The parameter *L*_ATP_ represents the unstressed length of the spring and is taken to be the step size of the head domain. Here, we use a fixed step size of 16 nm; however, in §5.4 we model variable step sizes in order to represent more faithfully experimental observations which show dynein stepping in a variable fashion. If MTBD D is in an unbound state and MTBD E is in a bound state, then the equations of motion can be shown to be given by

4.9 $$\begin{array}{rl} m_{S}\frac{d^{2}x_{D}}{dt^{2}} & =-\gamma_{\mathrm{ATP}}\frac{dx_{D}}{dt}-K_{\mathrm{ATP}}(x_{D}-p_{2k}-L_{\mathrm{ATP}})-\gamma_{S}\frac{dx_{D}}{dt}\\ & \;-K_{S}(x_{D}-x_{A}-L_{S}\cos(\theta_{\mathrm{AD}})), \end{array}$$

4.10 $$\;\frac{dx_{E}}{dt}=0,$$

for *t* ∈ [*t*_*i*_, *t*_*i*+1_] for i∈N such that 0 ≤ *t*_*i*_ < *t*_*i*+1_, where *p*_2*k*_ with $k\in N_{0}$ is the binding site that MTBD D was bound to at time *t* = *t*_*i*_. The equations are similar for when MTBD E is in the unbound state and MTBD D is in the bound state:

4.11 $$\;\frac{dx_{D}}{dt}=0,$$

4.12 $$\begin{array}{rl} m_{S}\frac{d^{2}x_{E}}{dt^{2}} & =-\gamma_{\mathrm{ATP}}\frac{dx_{E}}{dt}-K_{\mathrm{ATP}}(x_{E}-p_{2k+1}-L_{\mathrm{ATP}})-\gamma_{S}\frac{dx_{E}}{dt}\\ & \;-K_{S}(x_{E}-x_{B}-L_{S}\cos(\theta_{\mathrm{BE}})), \end{array}$$

again for *t* ∈ [*t*_*i*_, *t*_*i*+1_] and where *p*_2*k*+1_ with $k\in N_{0}$ is the binding site that MTBD E was bound to at time *t* = *t*_*i*_. Here, we are assuming some inherent coordination between the two MTBDs to keep the motor attached to the microtubule as one motor is unable to bind ATP, while the other is detached. The MTBDs are assumed to become unbound once the corresponding AAA+ ring binds ATP. This occurs randomly and the transition between states is explained below. The model is extended to include dwelling between steps, backward stepping and a variable step size in §5.4.

**Remark 4.2.**

Fixing the step size to 16 nm with predetermined binding sites is a strong assumption on where the MTBDs can bind. MTBDs are restricted to binding to specific binding sites on the microtubule due to the position of tubulin and cannot bind to a site that another MTBD is already bound to. The displacement of the MTBD, under a conformational change during the ATP cycle, has been suggested to be close to the 16 nm step size [19], with this being the predominant step size in these studies. Other step sizes have been recorded alongside off-axis displacement. For simplicity, we will consider the simplest model of dynein stepping in one space dimension. Variable steps sizes are considered in §5.4, while two-dimensional stepping is left for future studies.

### 4.1. Continual stochastic stepping

To model the continual stepping by dynein over a microtubule, stochasticity is introduced to the model via the randomness in which an AAA+ ring binds ATP and hence an MTBD becomes unbound. We assign the values *P*_*D*_ (*P*_*E*_) to the probability that MTBD E steps given that MTBD D (E) stepped previously and the maximum separation distance *d* that can occur between the MTBDs is defined. Consider *t* ∈ [0, *T*_Final_] with *T*_Final_ > 0 and *t*_*i*_ = *t*_*i*−1_ + *T*_Final_/*N* for *i* = 1, 2, …, *N*. Let **q** = {*q*_*i*_}_*i*=1:*N*_ be a random vector where *q*_*i*_ is from the uniform distribution on the interval (0, 1). If the maximum separation between the MTBDs has been exceeded, then it is assumed that the rearward head steps; else, given that MTBD *j* stepped previously, if *q*_*i*_ < *P*_*j*_, then MTBD E is set to be in the unbound state (i.e. unbound from the microtubule and undergoing the recovery stroke) and MTBD D is set to be in the bound state (i.e. bound to the microtubule). Otherwise, we assume that the MTBD D is in the unbound state and MTBD E in the bound state. Hence, we can define a step function *h*_*E*_ given by

4.13 $$h_{E}(t,\;x_{D},\;x_{E},\;d)=\left\{ \begin{array}{cc} 1 & \mathrm{if}\;x_{D}-x_{E}>d\;\mathrm{or}\;(q_{i}<P_{\;j}\;\mathrm{and}\;x_{E}-x_{D}\leq d),\\ 0 & \mathrm{otherwise}; \end{array}\right.$$

and similarly *h*_*D*_(*t*, *x*_*D*_, *x*_*E*_, *d*) = 1 − *h*_*E*_(*t*, *x*_*D*_, *x*_*E*_, *d*) for *t* ∈ [*t*_*i*_, *t*_*i*+1_] with *i* = 1, 2, …, *N*. This does assume some form of coordination between the two head domains of dynein as only one head will step during each time interval, but it does not enforce coordination of the stepping pattern itself if the head domains are allowed to separate past consecutive binding sites. The rearward head always steps if the two head domains become too far apart. This assumption reflects the existence of a linker that plays a critical role in gating dynein stepping behaviour thereby modelling tension-dependency at high interhead separation during the processive stepping [4]. In future studies, it might be worth introducing a model specifically taking into account how the linker gates dynein stepping behaviour [4]. The system of ODEs is therefore given by

4.14 $$m_{C}\frac{d^{2}x_{C}}{dt^{2}}=K_{C}(x_{T}-x_{C}-L_{C})-F_{C}-\gamma_{C}\frac{dx_{C}}{dt},$$

4.15 $$\begin{array}{rl} m_{T}\frac{d^{2}x_{T}}{dt^{2}} & =K_{T}(x_{B}-x_{T}-L_{T}\cos(\theta_{\mathrm{BT}}))-K_{T}(x_{T}-x_{A}-L_{T}\cos(\theta_{\mathrm{AT}}))\\ & \;-K_{C}(x_{T}-x_{C}-L_{C})-\gamma_{T}\frac{dx_{T}}{dt}, \end{array}$$

4.16 $$m_{M}\frac{d^{2}x_{A}}{dt^{2}}=K_{T}(x_{T}-x_{A}-L_{T}\cos(\theta_{\mathrm{AT}}))-K_{S}(x_{A}-x_{D}-L_{S}\cos(\theta_{\mathrm{AD}}))-\gamma_{M}\frac{dx_{A}}{dt},$$

4.17 $$m_{M}\frac{d^{2}x_{B}}{dt^{2}}=K_{S}(x_{E}-x_{B}-L_{S}\cos(\theta_{\mathrm{BE}}))-K_{T}(x_{B}-x_{T}-L_{T}\cos(\theta_{\mathrm{BT}}))-\gamma_{M}\frac{dx_{B}}{dt},$$

4.18 $$\begin{array}{rl} m_{S}h_{D}(t,\;x_{D},\;x_{E},\;d)\frac{d^{2}x_{D}}{dt^{2}} & =h_{D}(t,\;x_{D},\;x_{E},\;d)\left[-\gamma_{\mathrm{ATP}}\frac{dx_{D}}{dt}-K_{\mathrm{ATP}}(x_{D}-p_{2k}-L_{\mathrm{ATP}}\right)\\ & \;-K_{S}(x_{D}-x_{A}-L_{S}\cos(\theta_{\mathrm{AD}}))]-\gamma_{S}\frac{dx_{D}}{dt} \end{array}$$

4.19 $$\begin{array}{rl} \mathrm{and}\;\;m_{S}h_{E}(t,\;x_{D},\;x_{E},\;d)\frac{d^{2}x_{E}}{dt^{2}} & =h_{E}(t,\;x_{D},\;x_{E},\;d)\left[-\gamma_{ATP}\frac{dx_{E}}{dt}-K_{\mathrm{ATP}}(x_{E}-p_{2k+1}-L_{\mathrm{ATP}}\right)\\ & \;-K_{S}(x_{E}-x_{B}-L_{S}\cos(\theta_{\mathrm{BE}}))]-\gamma_{S}\frac{dx_{E}}{dt}, \end{array}$$

for *t* ∈ [0, *T*_Final_] with dimensional parameter values given in table 1 and the ranges or distributions for the stochastic parameters given in table 2.

**Table 2.** Stochastic stepping parameters and the respective ranges or distributions used in the mathematical model.

parameter	description	range/distribution
*d*	maximum separation distance between the MTBDs	8–80 nm
*μ*	mean dwell time	0–2 × 10^9^ ns
*P*_*D*_	probability that MTBD E steps given that MTBD D stepped previously	20–80%
*P*_*E*_	probability that MTBD E steps given that MTBD E stepped previously	20–80%
*P*_Back_	probability that the unbound MTBD steps backwards	0–20%
**q**	random vector that determines which MTBD steps	*U*(0, 1)
**q_D_**	random vector that determines when MTBD D steps	$\exp(\frac{1}{\mu })$
**q_E_**	random vector that determines when MTBD E steps	$\exp(\frac{1}{\mu })$
*n*	random number that determines the step size: — for a forward step of *nL*_ATP_ — for a backward step of −*nL*_ATP_	Pois(2) Pois(1)

**Remark 4.3.**

In this model, we only consider continual stepping; therefore, we fix the size of the time interval for each step, *T*_Step_ = *t*_*i*+1_ − *t*_*i*_, and hence *T*_Final_ will depend on the time interval *T*_Step_ and the number of steps *N*. Therefore, the stepping rate of the motors is predetermined. This assumption is relaxed in §§4.4 and 5.4 where independent and random dwell times are introduced to the model, respectively.

**Remark 4.4.**

The binding sites are predetermined. The initial binding site *p*_0_ is assigned a value and all binding sites are taken to be 8 nm away from the previous binding site. For each time step, the binding site is updated by taking the next binding site of the unbound MTBD. For example, if an MTBD is unbound on [*t*_*i*_, *t*_*i*+1_] and bound to *p*_*k*_ at time *t* = *t*_*i*_, then the binding site will be updated to *p*_*k*+2_ = (*p*_*k*_ + 16) nm for *t* = *t*_*i*+1_. It must be observed that the time continuity of the model is reflected and embedded in the time-variables associated with the domains. These variables are monitored (once computed) if they are located at the discrete steps or not. Variable step sizes are explored in §5.4; however, they are restricted to multiples of 8 nm to ensure that they can only bind at a specified binding site on the microtubule.

### 4.2. Non-dimensionalization

To non-dimensionalize the model, let *x*_C_ = *L*_C_*χ*_C_, *x*_T_ = *L*_T_*χ*_T_, *x*_*A*_ = *L*_*S*_*χ*_*A*_, *x*_*B*_ = *L*_*S*_*χ*_*B*_, *x*_*D*_ = *L*_*S*_*χ*_*D*_, *x*_*E*_ = *L*_*S*_*χ*_*E*_ and *t* = (*m*_C_/*γ*_C_)*τ*. The non-dimensionalized coefficients of the acceleration terms turn out to be small and the dynamics are dominated by the viscous drag [53]. Hence, neglecting the small coefficients of the second derivatives, we obtain the following non-dimensional system:

4.20 $$\;\alpha_{C}\frac{d\chi_{C}}{d\tau }=\left(\frac{1}{\rho_{1}}\chi_{T}-1\right)-\lambda -\chi_{C},$$

4.21 $$\alpha_{T}\frac{d\chi_{T}}{d\tau }=\left(\frac{1}{\rho_{2}}(\chi_{B}+\chi_{A})-\cos(\theta_{\mathrm{BT}})+\cos(\theta_{\mathrm{AT}})\right)+\rho_{1}\kappa_{1}(\chi_{C}+1)-(2+\kappa_{1})\chi_{T},$$

4.22 $$\;\alpha_{M}\frac{d\chi_{A}}{d\tau }=\rho_{2}\kappa_{2}(\chi_{T}-\cos(\theta_{\mathrm{AT}}))+(\chi_{D}+\cos(\theta_{\mathrm{AD}}))-(\kappa_{2+1})\chi_{A},$$

4.23 $$\;\alpha_{M}\frac{d\chi_{B}}{d\tau }=(\chi_{E}-\cos(\theta_{\mathrm{BE}}))+\rho_{2}\kappa_{2}(\chi_{B}+\cos(\theta_{\mathrm{BT}}))-(\kappa_{2}+1)\chi_{B},$$

4.24 $$\;\alpha_{S}\frac{d\chi_{D}}{d\tau }=h_{D}(\tau ,\;\chi_{D},\;\chi_{E},\;\delta )[\kappa_{3}(\beta_{2k}+\rho_{3})+(\chi_{A}+\cos(\theta_{\mathrm{AD}}))-(1+\kappa_{3})\chi_{D}]$$

4.25 $$\mathrm{and}\;\;\;\alpha_{S}\frac{d\chi_{E}}{d\tau }=h_{E}(\tau ,\;\chi_{D},\;\chi_{E},\;\delta )[\kappa_{3}(\beta_{2k+1}+\rho_{3})+(\chi_{B}+\cos(\theta_{\mathrm{BE}}))-(1+\kappa_{3})\chi_{E}].$$

The non-dimensional parameters are given by

$$\begin{array}{rl} \alpha_{C} & =\frac{\gamma_{C}\gamma_{C}}{m_{C}K_{C}},\;\alpha_{T}=\frac{\gamma_{T}\gamma_{C}}{m_{C}K_{T}},\;\alpha_{M}=\frac{\gamma_{M}\gamma_{C}}{m_{C}K_{S}},\;\alpha_{S}=\frac{(\gamma_{\mathrm{ATP}}+\gamma_{S})\gamma_{C}}{m_{C}K_{S}},\\ \rho_{1} & =\frac{L_{C}}{L_{T}},\;\rho_{2}=\frac{L_{T}}{L_{S}},\;\rho_{3}=\frac{L_{\mathrm{ATP}}}{L_{S}},\\ \kappa_{1} & =\frac{K_{C}}{K_{T}},\;\kappa_{2}=\frac{K_{T}}{K_{S}},\;\kappa_{3}=\frac{K_{\mathrm{ATP}}}{K_{S}},\\ \beta_{k} & =\frac{\;p_{k}}{L_{S}},\;\lambda =\frac{F_{C}}{K_{C}L_{C}},\;\delta =\frac{d}{L_{S}}. \end{array}$$

See table 1 for dimensional parameter values and table 2 for the range of values for *d*.

### 4.3. Initial conditions

We prescribe initial conditions as follows: MTBD D and E are taken to be at binding sites *p*_0_ and *p*_1_ = (*p*_0_ + 8) nm, respectively. The cargo is taken to be at the origin and the tail component is set to be at its natural length *L*_C_ from the cargo. The AAA+ rings are taken to be at the same point midway between the MTBDs, at a distance of the natural length *L*_T_ from the tail. Therefore, the initial conditions are set to be

4.26 $$\begin{array}{rl} & x_{C}(0)=0,\;x_{T}(0)=L_{C},\;x_{A}(0)=L_{C}+L_{T},\;x_{B}(0)=L_{C}+L_{T},\\ & x_{D}(0)=p_{0}=L_{C}+L_{T}-4,\;x_{E}(0)=p_{1}=L_{C}+L_{T}+4. \end{array}\}$$

The non-dimensional initial conditions are given by

4.27 $$\begin{array}{rl} & \chi_{C}(0)=0,\;\chi_{T}(0)=\rho_{1},\;\chi_{A}(0)=\rho_{2}+\rho_{1}\rho_{2},\;\chi_{B}(0)=\rho_{2}+\rho_{1}\rho_{2},\\ & \chi_{D}(0)=\beta_{0},\;\chi_{E}(0)=\beta_{1}. \end{array}\}$$

### 4.4. Independent stepping

It has been suggested in previous studies that interhead coordination is important to the stepping mechanism of two-headed cytoplasmic dynein [67]; therefore, we explore the significance of this coordination by considering the resultant behaviour if it is disrupted, i.e. if the two head domains step independently. The dwell time before the binding of ATP for each motor domain is modelled by the exponential distribution (see remark 4.5). We assume that these waiting times for each motor domain are independent of each other and are given by $q_{D}={\left\{q_{D}^{i}\right\}}_{i\in N}$ for MTBD D and $q_{E}={\left\{q_{E}^{i}\right\}}_{i\in N}$ for MTBD E with *q*^*i*^_*D*_ and *q*^*i*^_*E*_ taken from the exponential distribution with mean dwell time *μ*. The system continues to be modelled by equations (4.10)–(4.13), with different stepping functions to *h*_*D*_, *h*_*E*_ in equations (4.14) and (4.16). For MTBD D, we assume that it steps after *q*^*i*^_*D*_ ns, hence we define the following step function:

$$h_{q,D}(t,\;q_{D})=\left\{ \begin{array}{cc} 1 & \mathrm{if}\;t\in [t_{i}+q_{D}^{i},\;t_{i+1}]\\ 0 & \mathrm{if}\;t\in [t_{i},t_{i}+q_{D}^{i}], \end{array}\right.$$

where *t*_*i*_ and *t*_*i*+1_ are the times when MTBD D binds back onto the microtubule after stepping with *t*_0_ the initial time. The stepping function for MTBD E can be defined similarly:

$$h_{q,E}(t,\;q_{E})=\left\{ \begin{array}{cc} 1 & \mathrm{if}\;t\in [t_{\;j}+q_{E}^{i},\;t_{\;j+1}]\\ 0 & \mathrm{if}\;t\in [t_{\;j},t_{\;j}+q_{E}^{i}] \end{array}\right.$$

with *t*_*j*_ and *t*_*j*+1_ the times when MTBD E binds to the microtubule. Here, *t*_*j*_ denotes different time intervals to *t*_*i*_. Therefore, the following model system of ODEs can be derived as follows:

4.28 $$m_{C}\frac{d^{2}x_{C}}{dt^{2}}=K_{C}(x_{T}-x_{C}-L_{C})-F_{C}-\gamma_{C}\frac{dx_{C}}{dt},$$

4.29 $$\begin{array}{rl} m_{T}\frac{d^{2}x_{T}}{dt^{2}} & =K_{T}(x_{B}-x_{T}-L_{T}\cos(\theta_{\mathrm{BT}}))-K_{T}(x_{T}-x_{A}-L_{T}\cos(\theta_{\mathrm{AT}}))\\ & \;-K_{C}(x_{T}-x_{C}-L_{C})-\gamma_{T}\frac{dx_{T}}{dt}, \end{array}$$

4.30 $$m_{M}\frac{d^{2}x_{A}}{dt^{2}}=K_{T}(x_{T}-x_{A}-L_{T}\cos(\theta_{\mathrm{AT}}))-K_{S}(x_{A}-x_{D}-L_{S}\cos(\theta_{\mathrm{AD}}))-\gamma_{M}\frac{dx_{A}}{dt},$$

4.31 $$m_{M}\frac{d^{2}x_{B}}{dt^{2}}=K_{S}(x_{E}-x_{B}-L_{S}\cos(\theta_{\mathrm{BE}}))-K_{T}(x_{B}-x_{T}-L_{T}\cos(\theta_{\mathrm{BT}}))-\gamma_{M}\frac{dx_{B}}{dt},$$

4.32 $$\begin{array}{c} m_{S}h_{q,D}(t,\;q_{D})\frac{d^{2}x_{D}}{dt^{2}}=h_{q,D}(t,q_{D})[-\gamma_{\mathrm{ATP}}\frac{dx_{D}}{dt}-K_{\mathrm{ATP}}(x_{D}-p_{2k}-L_{\mathrm{ATP}})\\ -K_{S}(x_{D}-x_{A}-L_{S}\cos(\theta_{\mathrm{AD}}))]-\gamma_{S}\frac{dx_{D}}{dt} \end{array}$$

4.33 $$\begin{array}{c} \mathrm{and}\;m_{S}h_{q,E}(t,\;q_{E})\frac{d^{2}x_{E}}{dt^{2}}=h_{q,E}(t,q_{E})[-\gamma_{\mathrm{ATP}}\frac{dx_{E}}{dt}-K_{\mathrm{ATP}}(x_{E}-p_{2k+1}-L_{\mathrm{ATP}})\\ -K_{S}(x_{E}-x_{B}-L_{S}\cos(\theta_{\mathrm{BE}}))]-\gamma_{S}\frac{dx_{E}}{dt}, \end{array}$$

for *t* ∈ [0, *T*_Final_]. See table 1 for dimensional parameter values and table 2 for the ranges and distributions for the stochastic parameters.

**Remark 4.5.**

Experimental observations suggest that the dwell times of dynein can be approximated well by an exponential distribution with an average dwell time of 2 s, i.e. 2 × 10^9^ ns, per ATP cycle [48]. It is currently assumed that the dwell times are identical; however, differences in mean dwell times could be explored in future work.

We take a multiscale approach when non-dimensionalizing the model system, using one fast timescale for the stepping and one slow timescale for the dwelling. For the dwelling interval, we non-dimensionalize as above with *t*_*c*_ = *μ* and for the stepping intervals we take *t*_*c*_ = *m*_C_/*γ*_C_. For the sake of brevity, details of the non-dimensionalization are omitted here (see the electronic supplementary material for details).

## 5. Numerical experiments

The scheme is implemented in MATLAB for *N* stepping intervals of $\left[0,{\bar{T}}_{\mathrm{Final}}\right]$ with non-dimensional end time ${\bar{T}}_{\mathrm{Final}}=10^{8}$ and *N* = 100 using the solver *ode45* [68]. *ode45* is one of several solvers for integrating a system of non-stiff ordinary differential equations given appropriate initial conditions and is based on Runge–Kutta time-integrators. For further detailed description and implementation, we refer the interested reader to consult MATLAB MathWorks [68]. The initial conditions are given by

5.1 $$\begin{array}{rl} & \chi_{C}(0)=0,\;\chi_{T}(0)=\rho_{1},\;\chi_{A}(0)=\rho_{2}+\rho_{1}\rho_{2},\;\chi_{B}(0)=\rho_{2}+\rho_{1}\rho_{2},\\ & \chi_{D}(0)=\beta_{0},\;\chi_{E}(0)=\beta_{1}. \end{array}\}$$

For the initial step, it is assumed that MTBD D is in an unbound state and MTBD E is in a bound state. For each following step, a random number *q*_*i*_ is generated from the uniform distribution on the interval (0, 1) and the initial conditions are given by the values from the previous simulation: *χ*_C_(*τ*_*i*_), *χ*_T_(*τ*_*i*_), *χ*_*A*_(*τ*_*i*_), *χ*_*B*_(*τ*_*i*_), *χ*_*D*_(*τ*_*i*_) and *χ*_*E*_(*τ*_*i*_). From here onwards, we refer to trajectories, the physical loci or paths taken by each *x*_*i*_(*t*), and these represent the distances travelled in time. Also these could be referred to as positions of the components as a function of time.

**Remark 5.1.**

In all our numerical simulations (unless stated otherwise), in the absence of explicit modelling of the tension generated by the linker to gate dynein stepping behaviour, we impose a maximum interhead separation distance of 48 nm.

### 5.1. Stochastic stepping with limited coordination

**Figure 4.**
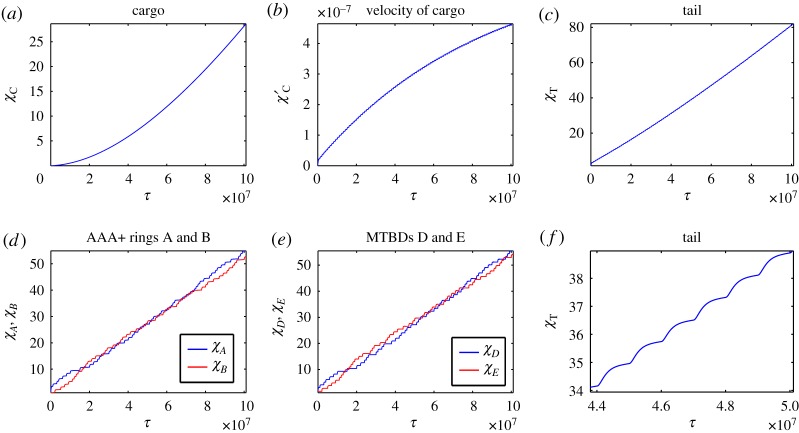
Numerical solutions to the model (4.17)–(4.23) with maximum separation between MTBDs at 48 nm and the probability that MTBD E steps at 50%. Plots over the whole time corresponding to (*a*) trajectory of the cargo, (*b*) velocity profile of the cargo, (*c*) trajectory of the tail domain, (*d*) trajectories of the AAA+ rings, (*e*) trajectories of the MTBDs and (*f*) trajectory of the tail domain for a representative subinterval.

**Figure 5.**
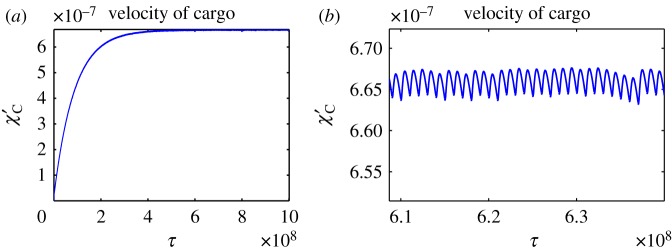
Numerical solutions to the model equations (4.17)–(4.23) with maximum separation between MTBDs at 48 nm and the probability that MTBD E steps at 50%, with end time *τ*_Final_ = 10^9^ and *N* = 1000. (*a*) Velocity profile of the cargo for *τ* ∈ [0, 10^9^]. (*b*) Velocity profile of the cargo for a representative subinterval illustrating the long-time dynamics of the cargo.

Initially, we assume that the motor domains bind ATP at random when they are both attached to the microtubule; therefore, we take *P*_*D*_ = *P*_*E*_ = 50%. This entails that the two head domains will not be highly coordinated in terms of their ATPase cycle, although they will experience some coordination, in terms of attachment to the microtubule, as we assume that only one motor domain can detach at a time. The results show a mixed stepping pattern for both the MTBDs and AAA+ rings with both not passing and passing stepping patterns present (figure 4*d*,*e*). This matches experimental observations (one-dimensional projections of two-dimensional experiments) on yeast cytoplasmic dynein, labelled at the AAA+ rings [36,37]. Here, we are able to compute the trajectories of the AAA+ rings and MTBDs, which is not yet achievable in experiments, as tagging functional MTBDs is technically challenging. The tail domain also moves with a stepping profile, as seen in experiments on dynein labelled at the tail domain (figure 4*c*,*f*). The cargo moves along the microtubule with increasing velocity, which becomes oscillatory at longer times once the dynein has settled into a stepping behaviour (figure 4*a*,*b*). By computing the solutions over a larger interval, with end time *τ*_Final_ = 10^9^ and *N* = 1000, the velocity of the cargo reaches a relative plateau where it stops increasing over time and oscillates within a small band (figure 5*a*,*b*), matching observations by Garrett *et al.* [10]. The velocity of the cargo increases over time before reaching a plateau due to the fact that dynein starts to move from a stationary position and therefore must pick up speed. We do not impose an external force at the initial phase of the stepping process.

**Figure 6.**
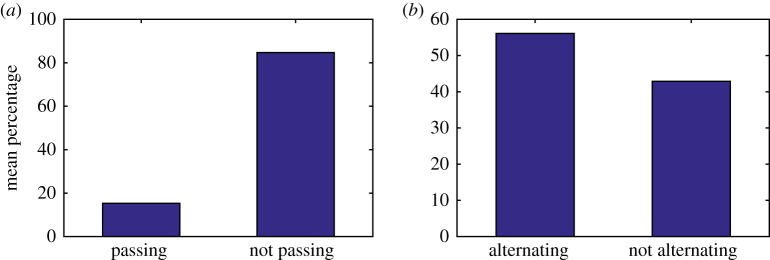
Bar charts showing the mean percentage of steps: (*a*) passing versus not passing and (*b*) alternating versus not alternating. The data represent the results of 1000 simulations with the probability that MTBD E steps set at 50% and the maximum separation distance set to be 48 nm.

In order to make statistical comparisons with experimental observations, we compiled data from 1000 simulations of the model and then took averages over these different realizations. Observations by Qiu *et al.* [37] show that approximately 83% of steps did not pass each other and in our simulations we have an average of 84.71% steps not passing when *d* = 48 nm (figure 6*a*, table 3). However, experimental results also show that dynein moves with predominately an alternating stepping pattern with approximately 74% of steps alternating in time [37], whereas our simulations show only 56.11% of steps alternating in time for *d* = 48 nm (figure 6*b*, table 3). This may be due to the randomness in the model where the probability of stepping is independent of which head stepped previously.

**Table 3.** Mean percentage of not passing steps and alternating steps given a range of values for the maximum separation distance *d* (nm). The data represent the results of 1000 simulations with the probability that MTBD E steps set at 50%. If *x*% of steps are not passing, then (100 − *x*)% of steps are passing. Similarly, if *x*% of steps are alternating, then (100 − *x*)% of steps are not alternating.

*d* (nm)	% not passing steps	% alternating steps
16	66.07	66.13
32	79.36	59.19
48	84.71	56.11
64	87.71	54.44

**Remark 5.2.**

Many experiments on cytoplasmic dynein, including the experiments by DeWitt *et al.* [36] and Qiu *et al.* [37], use dimerized yeast dynein. We have therefore also looked at a reduced version of the model for a dimerized dynein motor with no cargo and we get similar results for the stepping pattern and trajectories (see the electronic supplementary material for details).

### 5.2. Extensive interhead coordination

**Figure 7.**
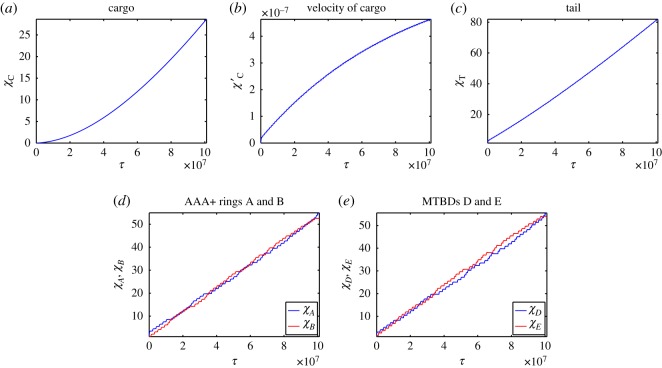
Numerical solutions to the model (4.17)–(4.23) with maximum separation between MTBDs at 48 nm and the probability that MTBD E steps set at 70% if the previous step was taken by MTBD D, and 30% otherwise. Plots over the whole time corresponding to (*a*) trajectory of the cargo, (*b*) velocity profile of the cargo, (*c*) trajectory of the tail domain, (*d*) trajectories of the AAA+ rings and (*e*) trajectories of the MTBDs.

**Figure 8.**
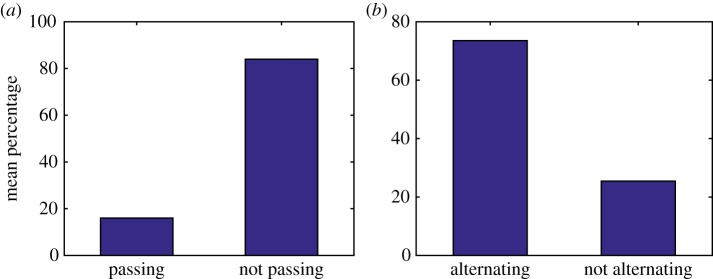
Bar charts showing the mean percentage of steps: (*a*) passing versus not passing and (*b*) alternating versus non-alternating. The data represent the results of 1000 simulations with the probability that MTBD E steps set at 70% if MTBD D stepped previously and 30% otherwise. The maximum separation distance is set to be 48 nm.

If dynein uses a more extensive form of interhead coordination, the probability that each MTBD steps will depend on the previous step. Therefore, the impact of dependent stepping probabilities on the model is investigated by taking *P*_*D*_ ≠ *P*_*E*_. It is assumed that the probability that MTBD E steps increases if MTBD D stepped previously and decreases if MTBD E stepped previously. By taking *P*_*D*_ = 70% and *P*_*E*_ = 30%, the results show the same mode of stepping to previous results, with a mixed stepping pattern of 84.0% not passing steps reflecting experimental observations of 83% (figures 7 and 8*a*). However, in comparison to our previous results, these results also resemble experimental observations with 73.5% of steps alternating in our simulations and 74% in experiments (figure 8*b*). This suggests that some form of coordination, in relation to the ATP cycles of each head domain, occurs between the motor domains of dynein, with one domain being more likely to step if the previous step was taken by the other motor domain. The proportion of alternating steps increases with an increase in the probability that MTBD E steps given that MTBD D stepped previously (table 4).

**Table 4.** Mean percentage of not passing steps and alternating steps given a range of values for the stepping probabilities of MTBD E. The data represent the results of 1000 simulations with the maximum separation distance set to be 48 nm. Observe that the percentage of not passing steps is independent of the probabilities *P*_*D*_ and *P*_*E*_, while the percentage of alternating steps is closely related. If *x*% of steps are not passing, then (100 − *x*)% of steps are passing. Similarly, if *x*% of steps are alternating, then (100 − *x*)% of steps are not alternating.

*P*_*D*_ (%)	*P*_*E*_ (%)	% not passing steps	% alternating steps
20	80	85.19	31.00
30	70	85.14	39.53
40	60	84.70	48.09
60	40	84.65	64.72
70	30	83.98	73.54
80	20	83.40	85.87

### 5.3. Independent stepping

**Figure 9.**
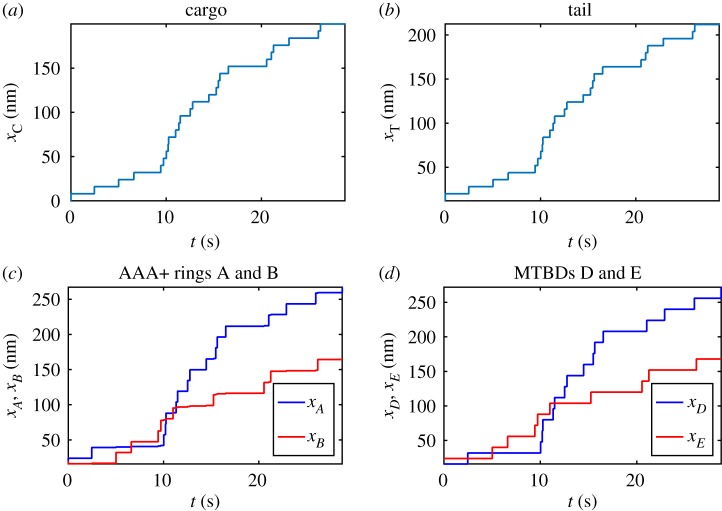
Dimensional numerical solutions to the model equations (4.26)–(4.31) with parameters *K*_ATP_ = 500 MDa ns^−2^, *γ*_ATP_ = 10 MDa ns^−1^ and all other parameters taken to be at their primary values given in table 1. Plots over the whole time corresponding to (*a*) trajectory of the cargo, (*b*) trajectory of the tail domain, (*c*) trajectories of the AAA+ rings and (*d*) trajectories of the MTBDs.

**Figure 10.**
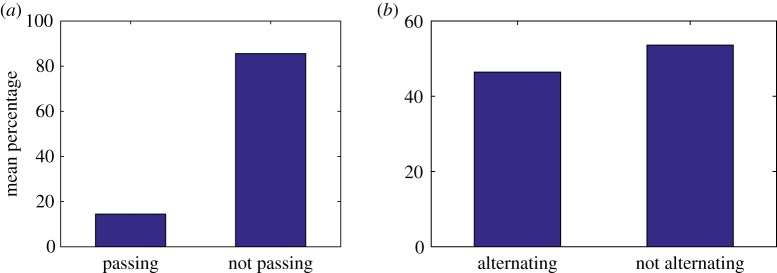
Bar charts showing the mean percentage of steps: (*a*) passing versus not passing and (*b*) alternating versus not alternating. The data represent the results of 100 simulations with *K*_ATP_ = 500 MDa ns^−2^ and *γ*_ATP_ = 10 MDa ns^−1^.

We now relax the assumption that there is coordination between the head domains and that they step independently. Owing to the independence of the two MTBDs, both MTBDs could become detached from the microtubule, if this occurs then the simulation is terminated and the number of steps and the run length are recorded. Initially, we consider forward stepping with a fixed step size of 16 nm. We consider a maximum of *N* = 100 steps with a mean dwell time of *μ* = 2 × 10^9^ ns for each head domain. Numerical simulations are run in MATLAB using the solver *ode15s* for the dwelling period and *ode45* for the stepping intervals [68]; example profiles are given in figure 9, and the mean percentage of steps passing and alternating are given in figure 10. The MATLAB solver *ode15s* is employed here to provide numerical solutions to a system of stiff ordinary differential equations (and in practice as well as systems of differential-algebraic equations (DAEs)), unlike *ode45* previously used [68].

By analysing the stepping behaviour of this model, we see that for larger values of *K*_ATP_, in the 400–1000 MDa ns^−2^ range in table 5, we achieve 83.63% to 86.97% not passing steps on average, which is close to the 83% seen in experiments. However, all values of *K*_ATP_ in table 5 give much lower values for the average percentage of alternating steps than those seen experimentally (predominantly around 49% compared to 74%). This suggests that this independent form of stepping cannot account for the alternating stepping patterns seen in experiments and hence there must be some form of coordination acting between the two head domains to account for this behaviour.

**Table 5.** Mean percentage of not passing and alternating steps, and mean number of steps in a run given a range of values for the parameters *K*_ATP_ (MDa ns^−2^) and *γ*_ATP_ (MDa ns^−1^). The data represent the results of 100 simulations with a mean dwell time of *μ* = 2 × 10^9^ ns. If *x*% of steps are not passing, then (100 − *x*)% of steps are passing. Similarly, if *x*% of steps are alternating, then (100 − *x*)% of steps are not alternating; except for the case labelled* where the number of steps in a run was always less than or equal to one and hence neither alternating or non-alternating steps were present.

*K*_ATP_ (MDa ns^−2^)	*γ*_ATP_ (MDa ns^−1^)	% not passing steps	% alternating steps	mean number of steps
10	10	100	0*	0.36
100	10	100	33.60	0.75
250	10	46.80	66.08	9.56
400	10	83.63	43.89	29.28
500	10	85.56	46.40	33.50
550	10	85.19	50.49	33.37
600	10	86.97	49.22	30.99
750	10	86.39	50.40	27.65
1000	10	84.56	48.07	22.92
500	1	85.06	47.12	32.90
500	10	85.56	46.40	33.50
500	100	84.52	43.58	26.71
500	1000	78.55	46.11	14.84

**Remark 5.3.**

We note that by allowing the head domains to step independently, they are able to diverge considerably implying larger interhead separation distances which might not be biologically realistic (e.g. figure 9*c*,*d*). This shows the need to explicitly model the linker which has been shown to gate interhead coordination at larger separation distances (and therefore able to bring the domains closer to each other) [4]. Alternatively, modelling internal forces that are known to influence the stepping behaviour may bring the complex back together [47]. Both of these remedies are the subject of our future studies.

Variations in the values of *K*_ATP_ show that run lengths are highly dependent on this parameter. We see that for *K*_ATP_ = 10 MDa ns^−2^ and *K*_ATP_ = 100 MDa ns^−2^ the mean number of steps in a run is less than one, suggesting that predominantly the run is terminated before the first step can be completed. Processivity is therefore dependent on the value of *K*_ATP_. Although this parameter cannot be directly measured in experiments as it is an approximation of the effects of the ATP force, it suggests that if the ATP cycle of the detached head domain is not completed quickly enough, then an uncoordinated detachment of the attached MTBD is likely to occur and hence the run will be terminated after fewer steps. Taking *K*_ATP_ = 500 MDa ns^−2^ gives a mean number of steps of 33.50 and mean run lengths of 275.95 nm for the cargo and 276.69 nm for the tail domain (tables 5 and 6). Although this gives the highest run lengths, these values are still much lower than those seen in experiments, with typical run lengths of 800 nm and 1.5 μm measured for murine and bovine dynein *in vitro* [69]. This suggests that although some processivity can be achieved with independent head domains, coordination is important to obtain the higher run lengths that are seen in experiments. The fact that the mean run lengths are lower than those seen in experiments could be due to the regulatory functions of dynactin and other cargo adaptor proteins such as BICD2 present *in vivo*, which activate long-distance movement of the motor [33,34,42]. Further modelling in this direction might help to confirm or refute such hypotheses. Variations in *γ*_ATP_ have little effect on the percentage of not passing and alternating steps; however, they do have an effect on run length, with an increase in *γ*_ATP_ > 10 MDa ns^−1^ leading to a fall in the mean number of steps and lower run length for all variables (tables 5 and 6).

**Table 6.** Mean run lengths for the cargo and tail domain given a range of values for the parameters *K*_ATP_ (MDa ns^−2^) and *γ*_ATP_ (MDa ns^−1^). The data represent the results of 100 simulations with a mean dwell time of *μ* = 2 × 10^9^ ns. If *x*% of steps are not passing, then (100 − *x*)% of steps are passing. Similarly, if *x*% of steps are alternating, then (100 − *x*)% of steps are not alternating.

*K*_ATP_ (MDa ns^−2^)	*γ*_ATP_ (MDa ns^−1^)	cargo (nm)	tail (nm)
10	10	10.96	15.54
100	10	14.09	19.12
250	10	84.52	88.87
400	10	242.20	243.79
500	10	275.95	276.69
550	10	277.54	277.79
600	10	255.87	255.95
750	10	229.17	229.18
1000	10	191.34	191.34
500	1	271.15	271.69
500	10	275.95	276.69
500	100	221.65	222.60
500	1000	126.70	129.37

**Remark 5.4.**

We have extended this model framework to include random backward stepping and a variable step size, details are discussed below in §5.4. If independent stepping is assumed, then this leads to a reduction in run length to 158 nm for the cargo. This is likely to be due to the presence of backward steps shortening the run length. However, larger step sizes may also lead to an increase in detachment time for a single head within the model, increasing the likelihood that the other head will also detach.

### 5.4. Backward stepping, variable step size and large-scale dwelling

In this section, we extend our modelling framework to take into account the backward stepping, variable step sizes and large-scale dwelling of dynein. Previously, we used a fixed time interval *T*_Final_/*N* for the stepping of a single MTBD; however, the active stepping of the MTBD should end when the MTBD binds to the microtubule. Consider the interval $\left[t_{i},\;t_{i+1}]\subset [t_{i},\;t_{i}+t_{\max}\right]$ with

$$t_{i+1}=\min\{t\in [t_{i},\;t_{i}+t_{\max}]\;:\;x_{i}(t)\geq p_{k+2}\},$$

where *p*_*k*+2_ is the next binding site for the unbound MTBD *j* and $t_{\max}$ is the maximum potential length of the stepping interval. Hence, the total time spent stepping is given by $T_{F}=\sum_{k=1}^{N}(t_{i+1}-t_{i})$. In order to model dwelling over large timescales, we take a multiscale approach by using one timescale for stepping and one for dwelling; variable step sizes and backward stepping are also included in the model (see the electronic supplementary material for details).

The model is solved numerically in MATLAB for *N* = 100 steps using *ode45* for the stepping model and the stiff solver *ode15s* [68] for the dwelling model. The non-dimensional systems are solved and then converted back to dimensional results so that they can be presented together on the same timescale. The maximum length of the stepping time interval is taken to be $\tau_{\max}=10^{6}$. The primary values are taken as follows: the probability that MTBD E steps given that MTBD D (E) stepped previously is taken to be *P*_*D*_ = 84% (*P*_*E*_ = 16%), the maximum separation distance is taken to be 48 nm and the probability of backward stepping (*P*_Back_) is set to be 20%. The mean dwell time is taken to be *μ* = 2 × 10^9^ ns as experimental results have shown the average dwell time for dynein to be 2 s [48]. For each step, we take *n* from the Poisson distribution about 2 to give the step size *nL*_ATP_ for the forward step sizes and *n* from the Poisson distribution about 1 for the backward steps to give the step size −*nL*_ATP_ (see remark 5.5). We assume that zero steps are possible, but they are not counted toward alternating or non-alternating steps. See table 2 for the range of values or distributions used for the stochastic parameters.

**Remark 5.5.**

Note that the distribution used to obtain the step size *nL*_ATP_ could be obtained through analysis of the experimental data to give a more accurate representation of the step sizes of a particular dynein species. However, it could also be used to analyse the effect of different distributions on stepping behaviour and run lengths which is left for future studies.

**Figure 11.**
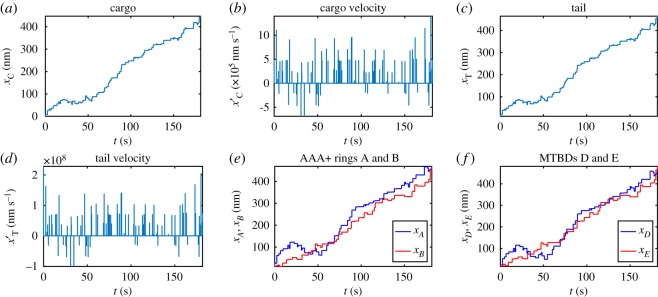
Dimensional numerical solutions to the model equations as described in §5.4 with maximum separation distance between MTBDs at 48 nm and the probability that MTBD E steps set at 84% if the previous step was taken by MTBD D, and 16% otherwise. The mean dwell time is taken to be 2 × 10^9^ ns and the probability of backward stepping is 20%. Plots over the whole time corresponding to (*a*) trajectory of the cargo, (*b*) velocity profile of the cargo, (*c*) trajectory of the tail domain, (*d*) velocity profile of the tail domain, (*e*) trajectories of the AAA+ rings and (*f*) trajectories of the MTBDs.

The results show similar profiles for the tail, AAA+ rings and MTBDs, with a clear presence of backward steps, variable step sizes and increased dwell times between steps; however, we see a significant difference for the velocity of the cargo (figure 11). Our computational results show that the frequency of alternating steps does not differ and is an emergent process of the modelling. On the other hand, our results show that the not-passing steps differed by approximately 1–4% for optimal parameters, which is not such a big variation. However, this variation becomes significant for small values of the maximum separation distance (see the electronic supplementary material for details). The cargo now dwells between steps, with an oscillatory velocity profile that returns to zero between steps which is similar to the *in vivo* experimental results shown by Garrett *et al.* [10]. Using the primary values for the parameters gives a maximum velocity of the cargo of 15 × 10^5^ nm s^−1^, and a velocity of up to 2 × 10^8^ nm s^−1^, for the tail domain. This is much higher than velocities measured experimentally with dynein typically moving at speeds of 600 nm s^−1^, at saturating ATP levels and at room temperature with *in vivo* velocities reaching up to 3 μm s^−1^ in mammalian neurons, although yeast dynein moves at slower speeds of around 50–80 nm s^−1^ [69]. A full parameter analysis of all unknown model parameters needs to be conducted in order to establish the parameter set which gives quantitatively accurate values for the velocity for each species and context.

Although the overall direction of travel for the AAA+ rings and MTBDs are closely related, we do see differences in their behaviour, with the two AAA+ rings being further apart from each other than the two MTBDs and crossing paths at different time points (figure 11*e*,*f*). This would suggest that labelling at the AAA+ ring may not automatically give a clear picture of the stepping behaviour at the MTBD. However, there is no significant difference in stepping pattern with 82.72% non-passing steps for the MTBDs and 83.15% for the AAA+ rings.

We also achieve a range of backward steps in the model. For a fixed step size and minimal dwelling of 2 ns (see remark 5.6), taking the probability of backward stepping to be 20% and the maximum separation distance to be 48 nm results in 24.91% backward steps, reflecting the experimental observations of Qiu *et al.* (23%) [37]. While in order to match the observations of Reck-Peterson *et al.* (13%), we can take the probability of backward stepping to be 10% and the maximum separation distance to be 56 nm to obtain 13.19% backward steps [48]. A probability of backward stepping of 0% does not mean that there will be no backward stepping in the model as we use this parameter to represent random backward stepping, which we differentiate from the corrective backward steps taken when the MTBDs are too far apart. We see from the results that this gives a very low presence of backward stepping, much lower than in experimental results. Hence, this suggests that the MTBDs might randomly step backwards or that tension within the complex causes restorative backward steps through some mechanism not explicitly modelled. However, such processes are beyond the scope of this study. Experimental studies have shown that dynactin plays an important role in the directionality of dynein, and hence we may need to explore these effects in greater detail [2,6,28–30,32,34,65]. The effects of stepping along the microtubule in two dimensions may play a role in backward stepping if the motor domain rotates due to the off-axis components of the steps. In our current model, we are setting external forces to be zero, but these forces may play a role in the directionality of the head domain for *in vivo* studies.

**Remark 5.6.**

The effects of backward stepping and variable step sizes were explored on an initial minimal dwelling model for a single timescale, using the non-dimensionalization given in §4.2, taking *μ* = 2 ns.

We explored variations in the maximum separation distance on the stepping patterns (table 7). The reduction in maximum separation distance increases the likelihood of backward stepping, this is to be expected as backward stepping is directly related to the separation distance in the model, with an unbound head stepping backwards if it is too far in front of the other MTBD. We also see that reducing the maximum separation distance increases the likelihood of passing steps, which makes sense as closer MTBDs are more likely to cross over one another during stepping.

**Table 7.** Mean percentage of not passing, alternating and backward steps given a range of values for the maximum separation distance *d* (nm). The data represent the results of 100 simulations with the probability that MTBD E steps set at 74% if MTBD D stepped previously and 26% otherwise. The probability of random backward stepping is set to be 10% and the mean dwell time is taken to be 2 ns. If *x*% of steps are not passing, then (100 − *x*)% of steps are passing. Similarly, if *x*% of steps are alternating, then (100 − *x*)% of steps are not alternating.

*d* (nm)	% not passing steps	% alternating steps	% backward steps
8	49.36	73.90	26.63
16	53.57	74.39	20.88
24	68.05	73.73	17.14
32	75.45	74.31	15.80
40	80.85	74.73	14.47
48	81.68	73.53	13.19
56	84.50	74.35	12.90
64	85.72	73.43	12.96
72	85.89	74.30	12.08
80	87.29	73.67	12.70

Increasing the stepping probability of MTBD E after MTBD D has stepped decreases the percentage of not passing steps and also decreases the percentage of backward steps (table 8). This is likely to occur as the increased coordination would create a more efficient stepping pattern reducing the prevalence of wasteful backward steps by keeping the motor domains closer together and hence passing steps would also be more likely to occur.

**Table 8.** Mean percentage of not passing, alternating and backward steps given a range of values for the stepping probabilities of MTBD E. The data represent the results of 100 simulations with the maximum separation distance set to be 56 nm. The probability of random backward stepping is set to be 10% and the mean dwell time is taken to be 2 ns. Observe that the % of not passing steps is independent of the probabilities *P*_*D*_ and *P*_*E*_, while the % of alternating steps is closely related. If *x*% of steps are not passing, then (100 − *x*)% of steps are passing. Similarly, if *x*% of steps are alternating, then (100 − *x*)% of steps are not alternating.

*P*_*D*_ (%)	*P*_*E*_ (%)	% not passing steps	% alternating steps	% backward steps
20	80	88.00	19.93	23.01
30	70	87.13	29.90	17.87
40	60	87.29	39.74	16.49
50	50	86.23	50.06	15.41
60	40	85.87	60.51	13.44
70	30	84.17	70.82	13.28
80	20	84.81	80.18	12.90

**Figure 12.**
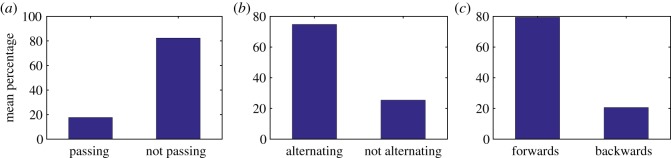
Bar charts showing the mean percentage of steps: (*a*) passing versus not passing, (*b*) alternating versus not alternating, (*c*) forwards versus backwards. The data represent the results of 1000 simulations with the probability that MTBD E steps set at 84% if MTBD D had stepped previously and 16% otherwise. The maximum separation distance is set to be 48 nm, the mean dwell time is 2 ns and the probability of random backward stepping is taken to be 20%.

**Figure 13.**
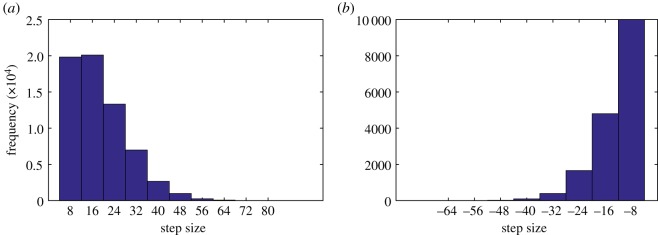
Histograms showing the distribution of step sizes (nm): (*a*) forward steps and (*b*) backward steps. The data represent the results of 1000 simulations with the probability that MTBD E steps set at 84% if MTBD D stepped previously and 16% otherwise. The maximum separation distance is set to be 48 nm, the mean dwell time is 2 ns and the probability of random backward stepping is taken to be 20%.

Owing to the presence of zero sized steps, in order to achieve similar results to experiments we take the probability that MTBD E steps set at 84% if the previous step was taken by MTBD D, and 16% otherwise. This results in 82.72% non-passing steps, 74.68% alternating steps and 20.91% backward steps (figure 12). Approximately 10% of steps by the MTBDs were of a zero step size, the majority of steps were of 8–16 nm and histograms of both forward and backward step distributions (not including zero steps) are given in figure 13. It is likely that dynein does experience step sizes of ‘zero’ length, i.e. detaches but rebinds to the same point on the microtubule. However, this stepping behaviour is not picked up (and therefore not accounted for) by the step-finding algorithms used in experimental data analysis. This suggests that the coordination between head domains could actually be higher in reality than recorded in experiments.

## 6. Discussion

In this study, we have derived a general integrative mechanistic model that describes the transport mechanism of cytoplasmic dynein. Our results give a mixed stepping pattern with a predominantly not passing stepping profile, which is an emergent process of the model, matching experimental observations. We have shown that there is likely to be some form of interhead coordination between the timing of the ATP cycles of the two AAA+ rings in order to account for the alternating patterns seen in experiments.

We have been able to model uncoordinated motion and have shown that dynein can still achieve some level of processivity through this mechanism. For example, the model achieves run lengths close to those seen for murine dynein in the absence of dynactin, when using a fixed forward stepping pattern. This may suggest that either dynactin has some influence on the coordination of the motor domains or that we need to account for the effect of dynactin in our model in some other way.

*Loa* dynein in mice has been shown to exhibit shorter run lengths than wild-type complexes [10–12]. Ori-McKenney *et al.* [11] measured run lengths of 259 nm for *Loa*+/− mutants and 175 nm for *Loa*−/− mutants; we are able to achieve similar run lengths through an appropriate choice of parameters. Ori-McKenney *et al.* [11] suggest that the *Loa* mutation may cause altered coordination in the motor domain of dynein. Our results suggest that it may be possible that this mutation disrupts the coordination within the complex, potentially leading to more frequent detachment of the motor from the microtubule and shorter run lengths. Deng *et al.* [14] have also shown that the *Loa* mutation causes dynein to have a lower affinity to dynactin, and so it may be through this disruption that the mutation affects the transport mechanisms of dynein.

It would be interesting to investigate the effect of the differences in these dwell times rather than assuming that the mean dwell time for each head domain is equivalent. In particular, allowing the lagging and leading head to have different dwell times may encourage a more coordinated stepping pattern, and experiments have shown that the lagging and leading heads have different stepping characteristics [36,37].

Currently, the motor domain can diverge as we assume that once the MTBD is bound, it is bound until a conformational change through ATP hydrolysis cycle occurs, it would therefore be interesting to introduce the effect of forces on detachment in this model. It has been shown by Gennerich *et al.* [47] that dynein can walk through applied force alone, and so these forces are important to the model. Another alternative is to introduce explicit modelling of the linker that has been shown to gate the ATP-dependent release of dynein from microtubules [4]. In particular, the linker has been shown to play a critical role in gating dynein stepping behaviour at high interhead separation distances. By introducing a linker, a tension-dependent force that acts to retract the leading head or to pull the lagging head will counterbalance the larger separation distances between heads during dynein stepping behaviour.

We have also been able to incorporate backward stepping, a variable step size and dwelling over large timescales into the model. The results give trajectories for the complex and cargo that qualitatively match experimental observations. Although we have compared our results qualitatively and quantitatively with results published in the literature, it would be beneficial to carry out detailed comparisons for a specific dynein whereby space–time series distributions data are provided. An ideal candidate is to employ a Bayesian parameter identification approach that allows us to compute optimal parameter distributions resulting from fitting the solution of the mathematical model (with all parameters assumed unknown) to experimental data (known) in an optimal sense [70]. The result of this approach is the rich statistical data that provide various statistical measures such as mean, variance and 95% credible regions. Furthermore, velocities can also be computed as distributions, which is more suitable for analysis and comparison to experiments. This approach forms part of our current studies, the only requirement is finding appropriate experimental data generated in terms of space–time series to allow us to optimize parameter identification such that the model solution best fits the data.

We have also shown that backward stepping that is directly related to the separation within the complex cannot account for the high percentages of backward stepping seen experimentally, and hence there must be something else external to this simple model causing these characteristics. We suggest that the impact of dynactin on the transport mechanisms and the three-dimensional nature of dynein need to be explored further with regard to their impact on backward stepping.

By prescribing the levels of coordination within the model, we can match experimental observations of the alternating stepping pattern, but when considering the possibility of ‘zero’ step sizes, this coordination must be higher than that seen in experimental observations. The model predicts the preference of dynein to the not passing stepping pattern when the motor is allowed to separate (which is realistic due to the large step sizes seen in experiments), and this matches experimental observations. The model also predicts that species of dynein which prefer a tighter conformation may be more likely to experience backward steps and have a higher prevalence of passing steps. Stronger coordination between the two motor domains could also reduce backward stepping, which leads to more efficient stepping as backward steps may be wasteful.

Other future works involve studies to investigate the effects of variable dwell times and strain-dependent stepping to establish a complete model which incorporates all aspects of transport mechanisms for cytoplasmic dynein. Apart from extending the model to take into account dynactin and tension linker domain, it will also be interesting to model multiple dyneins and how they aid or hinder the stepping behaviour. Recent studies by Urnavicius *et al.* [28] and Grotjahn *et al.* [29] reveal that dynactin has the capacity to recruit a team of dyneins for processive motility. However, to the best of our knowledge, no mathematical model has been formulated that could describe such experimental observations. The approach presented here sets foundations for such a study.

## Supplementary Material

Walking on microtubules: A mechanical model describing the stepping behaviour of cytoplasmic dynein

## Data accessibility

All data are provided in full in the results section of this paper. The numerical algorithms are available from the Dryad Digital Repository: https://doi.org/10.5061/dryad.1615h [71].
