# Supplementary material for: A mathematical understanding of how cytoplasmic dynein walks on microtubules
Source: R Soc Open Sci. 2018 Aug 8;5(8):171568. doi: 10.1098/rsos.171568 (PMC6124060; doi:10.1098/rsos.171568)
Supplement: Walking on microtubules: A mechanical model describing the stepping behaviour of cytoplasmic dynein [file rsos171568supp1.pdf]

Supplementary Material

Walking on microtubules: A mechanical model describing the stepping behaviour of cytoplasmic dynein

Laurie Trott[*], Majid Hafezparast[†§] and Anotida Madzvamuse[‡§]

1 Model for Dimerized Yeast with No Cargo

Many stepping experiments studying dynein are conducted using a dimerized form. To study a dimerized form of dynein a shorter form of dynein's tail is modelled by using two identical springs connecting the tail to each of the AAA+ rings, this tail can be shortened according to the specific dimerisation by the use of the parameter L_T. It is also assumed that there is no cargo. The Qdots in these experiments tend to be attached to the AAA+ rings themselves and it is assumed that the effect of these Qdots on the dynamics can be ignored. The model

[*]L. Trott: Dept. of Mathematics, School of Mathematical and Physical Sciences and School of Life Sciences, University of Sussex, Brighton, BN1 9QH, UK. Email: L.E.Trott@sussex.ac.uk

[†]M. Hafezparast: School of Life Sciences, University of Sussex, Brighton, BN1 9QG, UK. Tel.: 01273 678214. Email: M.Hafezparast@sussex.ac.uk

[‡]A. Madzvamuse: Dept. of Mathematics, School of Mathematical and Physical Sciences, University of Sussex, Brighton, BN1 9QH, UK. Tel.: 01273 873529. Email: A.Madzvamuse@sussex.ac.uk

[§]Correspondance to: M.Hafezparast@sussex.ac.uk and A.Madzvamuse@sussex.ac.uk.

equations (15)-(19) in the main paper can be modified to:

$$m_T \frac{d^2 x_T}{dt^2} = K_T\Big(x_B - x_T - L_T \cos(\theta_{BT})\Big) - K_T\Big(x_T - x_A - L_T \cos(\theta_{AT})\Big) - \gamma_T \frac{dx_T}{dt}, \tag{1}$$

$$m_M \frac{d^2 x_A}{dt^2} = K_T\Big(x_T - x_A - L_T \cos(\theta_{AT})\Big) - K_S\Big(x_A - x_D - L_S \cos(\theta_{AD})\Big) - \gamma_M \frac{dx_A}{dt}, \tag{2}$$

$$m_M \frac{d^2 x_B}{dt^2} = K_S\Big(x_E - x_B - L_S \cos(\theta_{BE})\Big) - K_T\Big(x_B - x_T - L_T \cos(\theta_{BT})\Big) - \gamma_M \frac{dx_B}{dt}, \tag{3}$$

$$\begin{aligned} m_S h_D(t, x_D, x_E, d) \frac{d^2 x_D}{dt^2} = h_D(t, x_D, x_E, d)\Big[&- \gamma_{ATP} \frac{dx_D}{dt} - K_{ATP}(x_D - p_{2k} - L_{ATP}) \\ &- K_S(x_D - x_A - L_S \cos(\theta_{AD}))\Big] - \gamma_S \frac{dx_D}{dt}, \end{aligned} \tag{4}$$

$$\begin{aligned} m_S h_E(t, x_D, x_E, d) \frac{d^2 x_E}{dt^2} = h_E(t, x_D, x_E, d)\Big[&- \gamma_{ATP} \frac{dx_E}{dt} - K_{ATP}(x_E - p_{2k+1} - L_{ATP}) \\ &- K_S(x_E - x_B - L_S \cos(\theta_{BE}))\Big] - \gamma_S \frac{dx_E}{dt}, \end{aligned} \tag{5}$$

for $t \in [0, T_{Final}]$ and with initial conditions:

$$x_T(0) = 0,\ x_A(0) = L_T,\ x_B(0) = L_T,$$

$$x_D(0) = p_0 = L_T - 4,\ x_E(0) = p_1 = L_T + 4.$$

The nondimensionalization is implemented similarly with a notable change for the time characteristic:

$$x_T = L_T \chi_T,\ x_A = L_S \chi_A,\ x_B = L_S \chi_B,\ x_D = L_S \chi_D,\ x_E = L_S \chi_E,\ t = \frac{m_T}{\gamma_T}\tau.$$

Hence, the nondimensional model system is given by the following ODEs:

$$\alpha_T \frac{d\chi_T}{d\tau} = \Big(\frac{1}{\rho_2}(\chi_B + \chi_A) - \cos(\theta_{BT}) + \cos(\theta_{AT})\Big) - 2\chi_T, \tag{6}$$

$$\alpha_M \frac{d\chi_A}{d\tau} = \rho_2\kappa_2\Big(\chi_T - \cos(\theta_{AT})\Big) + \Big(\chi_D + \cos(\theta_{AD})\Big) - (\kappa_2 + 1)\chi_A, \tag{7}$$

$$\alpha_M \frac{d\chi_B}{d\tau} = \Big(\chi_E - \cos(\theta_{BE})\Big) + \rho_2\kappa_2\Big(\chi_B + \cos(\theta_{BT})\Big) - (\kappa_2 + 1)\chi_B, \tag{8}$$

$$\alpha_S \frac{d\chi_D}{d\tau} = h_D(\tau, \chi_D, \chi_E, \delta)\Big[\kappa_3(\beta_{2k} + \rho_3) + \Big(\chi_A + \cos(\theta_{AD})\Big) - (1 + \kappa_3)\chi_D\Big], \tag{9}$$

$$\alpha_S \frac{d\chi_E}{d\tau} = h_E(\tau, \chi_D, \chi_E, \delta)\Big[\kappa_3(\beta_{2k+1} + \rho_3) + \Big(\chi_B + \cos(\theta_{BE})\Big) - (1 + \kappa_3)\chi_E\Big]. \tag{10}$$

The nondimensional parameters are given by

$$\alpha_T = \frac{\gamma_T^2}{m_T K_T}, \quad \alpha_M = \frac{\gamma_M \gamma_T}{m_T K_S}, \quad \alpha_S = \frac{(\gamma_{ATP} + \gamma_S)\gamma_T}{m_T K_S},$$
$$\rho_2 = \frac{L_T}{L_S}, \quad \rho_3 = \frac{L_{ATP}}{L_S},$$
$$\kappa_2 = \frac{K_T}{K_S}, \quad \kappa_3 = \frac{K_{ATP}}{K_S},$$
$$\beta_k = \frac{p_k}{L_S}, \quad \delta = \frac{d}{L_S}.$$

See Table 1 within the main paper for dimensional parameter values, with the range of values for d given in Table 2. The trajectories for the tail, AAA+ rings and MTBDs are similar to results from the full model (see Figure 1). The statistics for the stepping patterns are also similar with 84.63% not-passing steps and 56.23% alternating steps (see Figure 2).

2 Model with Variable Angles

If the assumption that the angles in the model are fixed is relaxed then we must solve a two-dimensional system. Let y_C, y_T, y_A, y_B, y_D and y_E represent the height of the cargo, tail, AAA+ rings A and B, and MTBDs D and E respectively. The only forces applied vertically are

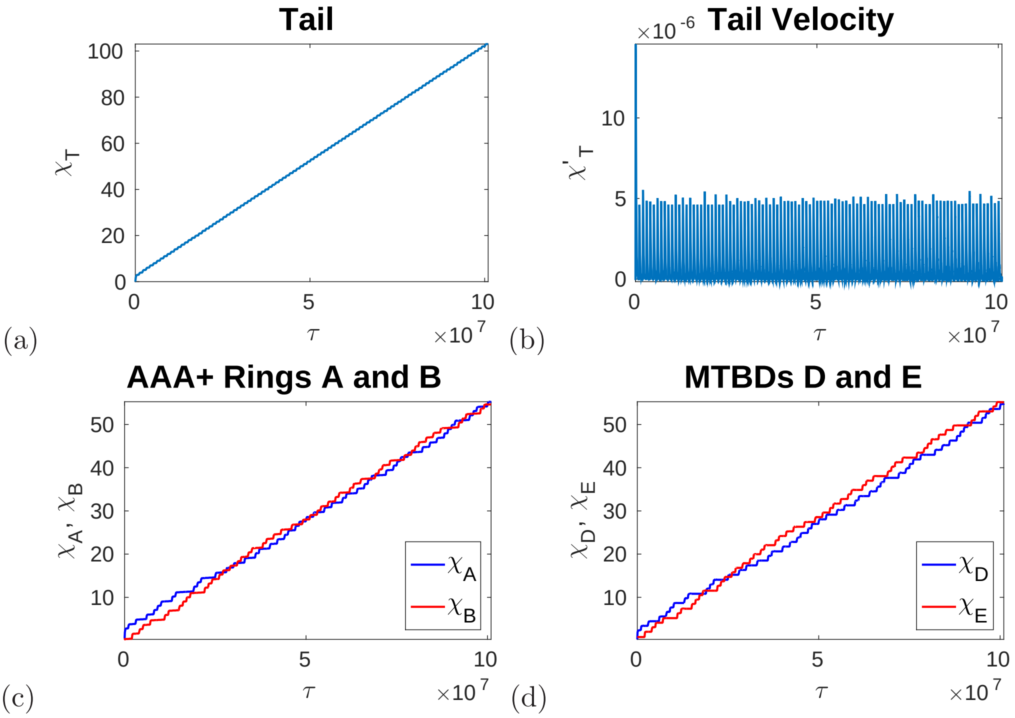

Figure 1: (Online version in colour.) Numerical solutions to the model equations (6) - (10) with maximum separation distance between MTBDs at 48 nm and the probability that MTBD E steps at 50%. Plots over the whole time corresponding to (a) trajectory of the tail, (b) velocity profile of the tail, (c) trajectories of the AAA+ rings, and (d) trajectories of the MTBDs.

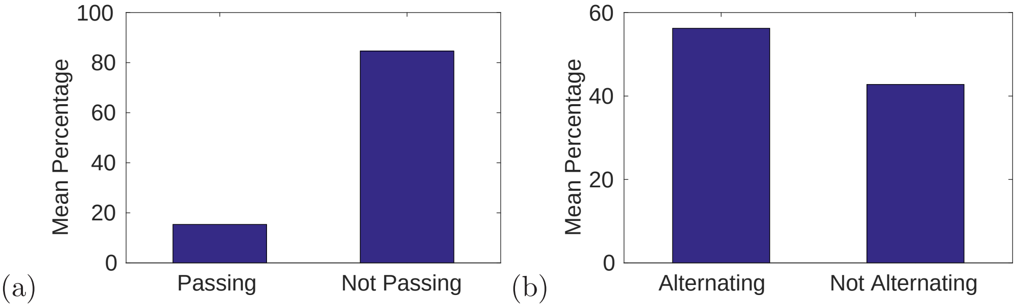

Figure 2: (Online version in colour.) Bar charts showing the mean percentage of steps: (a) passing vs not passing and (b) alternating vs non-alternating. The data represents the results of 1000 simulations with the probability that MTBD E steps set at 50%. The maximum separation distance is set to be 48 nm.

the spring forces and drag, by using Hooke's Law and Stokes' Law respectively and equating the mass multiplied by the acceleration to the net force we derive the following system of equations for the cargo, tail and AAA+ rings:

$$m_C \frac{d^2 y_C}{dt^2} = -K_C(y_C - y_T - L_C \sin(\theta_{CT})) - \gamma_C \frac{dy_C}{dt}, \tag{11}$$

$$\begin{aligned} m_T \frac{d^2 y_T}{dt^2} &= K_C(y_C - y_T - L_C \sin(\theta_{CT})) - K_T(y_T - y_B - L_T \sin(\theta_{BT})) \\ &\quad - K_T(y_T - y_A - L_T \sin(\theta_{AT})) - \gamma_T \frac{dy_T}{dt}, \end{aligned} \tag{12}$$

$$m_A \frac{d^2 y_A}{dt^2} = K_T(y_T - y_A - L_T \sin(\theta_{AT})) - K_S(y_A - y_D - L_S \sin(\theta_{AD})) - \gamma_A \frac{dy_A}{dt}, \tag{13}$$

$$m_B \frac{d^2 y_B}{dt^2} = K_T(y_T - y_B - L_T \sin(\theta_{BT})) - K_S(y_B - y_E - L_S \sin(\theta_{BE})) - \gamma_B \frac{dy_B}{dt}, \tag{14}$$

and angles given by:

$$\theta_{CT} = \arctan\left(\frac{y_C - y_T}{x_T - x_C}\right), \ \theta_{AT} = \arctan\left(\frac{y_T - y_A}{x_T - x_A}\right), \ \theta_{BT} = \arctan\left(\frac{y_T - y_B}{x_B - x_T}\right)$$

$$\theta_{AD} = \arctan\left(\frac{y_A - y_D}{x_D - x_A}\right), \ \theta_{BE} = \arctan\left(\frac{y_B - y_E}{x_E - x_B}\right)$$

See Table 1 within the main paper for dimensional parameter values. For the MTBDs it is assumed that they do not move away from the microtubule. This is a valid assumption as whilst attached, the MTBDs will not move vertically and biophysical models have predicted that the movement of the MTBDs under the action of ATP hydrolysis is one-dimensional along the microtubule [31]. Therefore:

$$y_D = 0, \tag{15}$$

$$y_E = 0. \tag{16}$$

The vertical components are nondimensionalized as following: $y_C = L_C \psi_C$, $y_T = L_T \psi_T$, $y_A = L_S \psi_A$, $y_B = L_S \psi_B$, $y_D = L_S \psi_D$, $y_E = L_S \psi_E$ and $t = \frac{m_C}{\gamma_C} \tau$ and acceleration is assumed to be

small. Therefore, the following system of nondimensional ODEs is obtained:

$$\alpha_C \frac{d\psi_C}{d\tau} = -(\psi_C - \frac{1}{\rho_1}\psi_T - \sin(\phi_{CT})), \tag{17}$$

$$\alpha_T \frac{d\psi_T}{d\tau} = \kappa_1(\rho_1\psi_C - \psi_T - \rho_1 \sin(\phi_{CT})) - (\psi_T - \frac{1}{\rho_2}\psi_B - \sin(\phi_{BT})) - (\psi_T - \frac{1}{\rho_2}\psi_A - \sin(\phi_{AT})), \tag{18}$$

$$\alpha_M \frac{d\psi_A}{d\tau} = \kappa_2(\rho_2\psi_T - \psi_A - \rho_2 \sin(\phi_{AT})) - (\psi_A - \psi_D - \sin(\phi_{AD})), \tag{19}$$

$$\alpha_M \frac{d\psi_B}{d\tau} = \kappa_2(\rho_2\psi_T - \psi_B - \rho_2 \sin(\phi_{BT})) - (\psi_B - \psi_E - \sin(\phi_{BE})), \tag{20}$$

$$\frac{d\psi_D}{d\tau} = 0, \tag{21}$$

$$\frac{d\psi_E}{d\tau} = 0; \tag{22}$$

where

$$\alpha_C = \frac{\gamma_C\gamma_C}{m_C K_C}, \quad \alpha_T = \frac{\gamma_T\gamma_C}{m_C K_T}, \quad \alpha_M = \frac{\gamma_M\gamma_C}{m_C K_S},$$
$$\rho_1 = \frac{L_C}{L_T}, \quad \rho_2 = \frac{L_T}{L_S},$$
$$\kappa_1 = \frac{K_C}{K_T}, \quad \kappa_2 = \frac{K_T}{K_S}.$$

and the angles are given by:

$$\phi_{CT} = \arctan\left(\frac{\rho_1\psi_C - \psi_T}{\chi_T - \rho_1\chi_C}\right),\ \phi_{AT} = \arctan\left(\frac{\rho_2\psi_T - \psi_A}{\rho_2\chi_T - \chi_A}\right),\ \phi_{BT} = \arctan\left(\frac{\rho_2\psi_T - \psi_B}{\chi_B - \rho_2\chi_T}\right),$$
$$\phi_{AD} = \arctan\left(\frac{\psi_A - \psi_D}{\chi_D - \chi_A}\right),\ \phi_{BE} = \arctan\left(\frac{\psi_B - \psi_E}{\chi_E - \chi_B}\right).$$

Initial conditions are given by

$$\psi_C(0) = \frac{1}{\rho_1} sin(\phi_{BT}(0)) + \frac{1}{\rho_1\rho_2} sin(\phi_{BE}(0)),\ \psi_T(0) = sin(\phi_{BT}(0)) + \frac{1}{\rho_2} sin(\phi_{BE}(0)),$$
$$\psi_A(0) = sin(\phi_{AD}(0)),\ \psi_B(0) = sin(\phi_{BE}(0)),\ \psi_D(0) = 0,\ \psi_E(0) = 0.$$

with angles given by:

$$\phi_{CT}(0) = \pi,\ \phi_{AT}(0) = \frac{33\pi}{180},\ \phi_{BT}(0) = \frac{33\pi}{180},\ \phi_{AD}(0) = \frac{53\pi}{180},\ \phi_{BE}(0) = \frac{53\pi}{180}.$$

The results show very similar trajectories for the cargo, tail, AAA+ rings and MTBDs for the horizontal motion (see Figure 3). The statistics for the stepping pattern are also very similar with 86.57% non-passing steps and 55.65% alternating steps (see Figure 4). Allowing the angles to vary to such an extent may not be biologically realistic and so further work needs to be carried out in order to model the changes in these angles appropriately. However, these preliminary results do suggest that the assumption on fixed angles in the current model is unlikely to affect our results negatively.

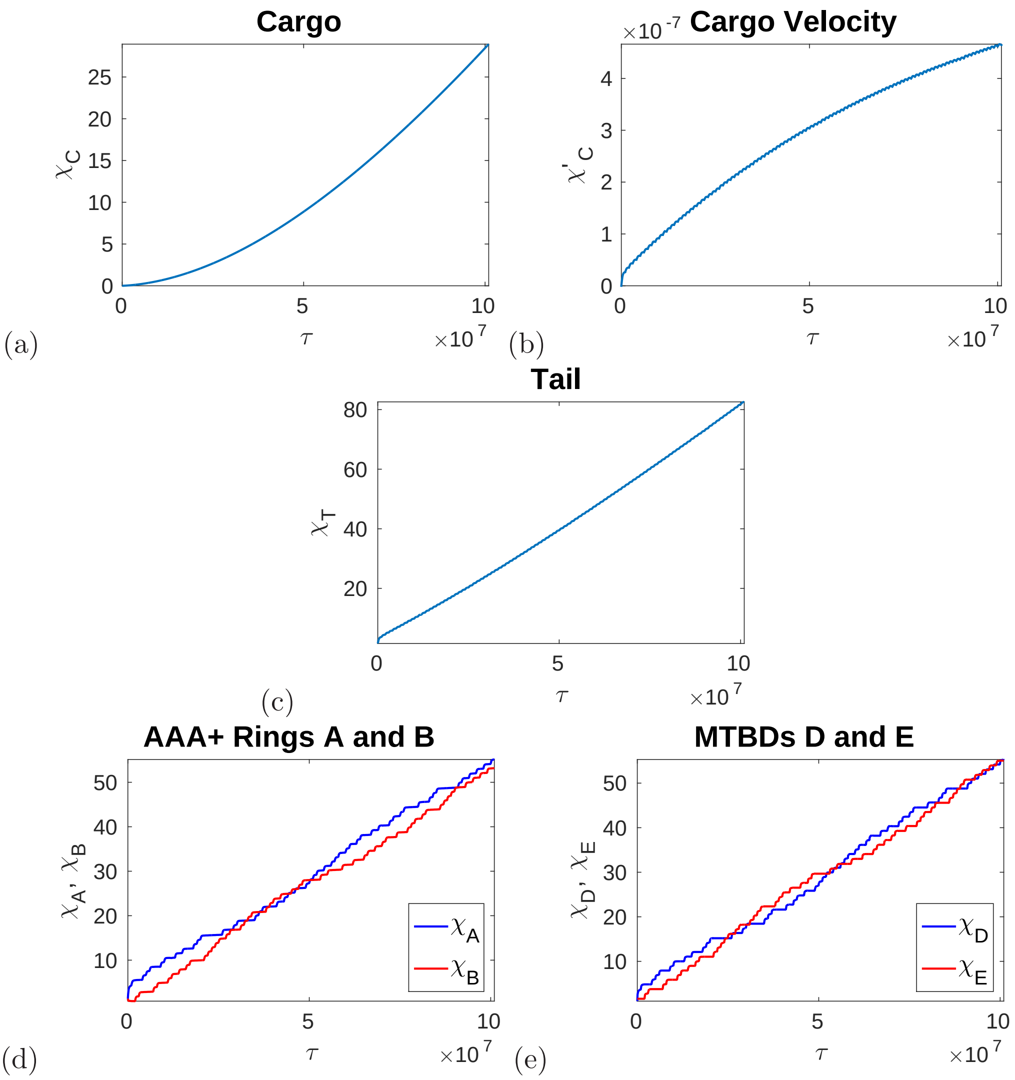

Figure 3: (Online version in colour.) Numerical solutions to the model equations (17) - (22) and (20) - (25) from the main paper, with maximum separation distance between MTBDs at 48 nm and the probability that MTBD E steps at 50%. Plots over the whole time corresponding to (a) trajectory of the cargo, (b) velocity profile of the cargo, (c) trajectory of the tail domain, (d) trajectories of the AAA+ rings, and (e) trajectories of the MTBDs.

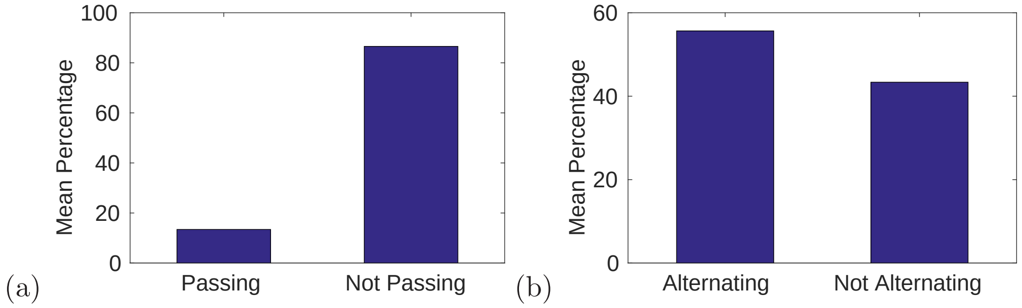

Figure 4: (Online version in colour.) Bar charts showing the mean percentage of steps: (a) passing vs not passing and (b) alternating vs non-alternating. The data represents the results of 1000 simulations with the probability that MTBD E steps set at 50%. The maximum separation distance is set to be 48 nm.

3 Alternate Angles: Parallel Stalks

In order to reflect observations from structural studies, the model can be altered to ensure that the stalks are parallel to each other (see Figure 5). The revised system of ODEs is given by:

$$m_C \frac{d^2 x_C}{dt^2} = K_C\Big(x_T - x_C - L_C\Big) - F_C - \gamma_C \frac{dx_C}{dt}, \tag{23}$$

$$\begin{aligned} m_T \frac{d^2 x_T}{dt^2} &= K_T\Big(x_B - x_T - L_T\cos(\theta_{BT})\Big) - K_T\Big(x_T - x_A - L_T\cos(\theta_{AT})\Big) \\ &\quad - K_C\Big(x_T - x_C - L_C\Big) - \gamma_T \frac{dx_T}{dt}, \end{aligned} \tag{24}$$

$$m_M \frac{d^2 x_A}{dt^2} = K_T\Big(x_T - x_A - L_T\cos(\theta_{AT})\Big) + K_S\Big(x_D - x_A - L_S\cos(\theta_{AD})\Big) - \gamma_M \frac{dx_A}{dt}, \tag{25}$$

$$m_M \frac{d^2 x_B}{dt^2} = K_S\Big(x_E - x_B - L_S\cos(\theta_{BE})\Big) - K_T\Big(x_B - x_T - L_T\cos(\theta_{BT})\Big) - \gamma_M \frac{dx_B}{dt}, \tag{26}$$

$$\begin{aligned} m_S h_D(t, x_D, x_E, d) \frac{d^2 x_D}{dt^2} &= h_D(t, x_D, x_E, d)\Big[- \gamma_{ATP} \frac{dx_D}{dt} - K_{ATP}(x_D - p_{2k} - L_{ATP}) \\ &\quad - K_S(x_D - x_A - L_S\cos(\theta_{AD}))\Big] - \gamma_S \frac{dx_D}{dt}, \end{aligned} \tag{27}$$

$$\begin{aligned} m_S h_E(t, x_D, x_E, d) \frac{d^2 x_E}{dt^2} &= h_E(t, x_D, x_E, d)\Big[- \gamma_{ATP} \frac{dx_E}{dt} - K_{ATP}(x_E - p_{2k+1} - L_{ATP}) \\ &\quad - K_S(x_E - x_B - L_S\cos(\theta_{BE}))\Big] - \gamma_S \frac{dx_E}{dt}, \end{aligned} \tag{28}$$

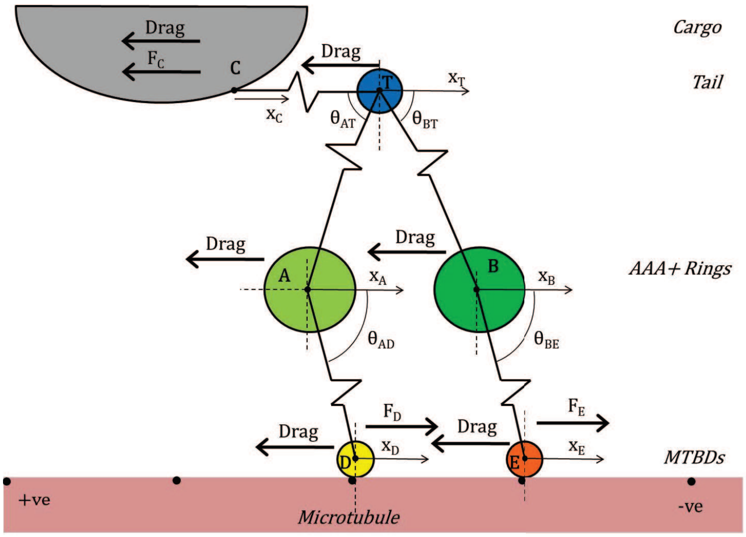

Figure 5: (Online version in colour.) A schematic diagram of the mechanical model with stalk angles parallel (adapted from [29]). The cargo is modelled as a sphere (grey) and regulators of binding to dynein are modelled as part of this cargo. The binding of the cargo to the tail domain is modelled by a spring. The tail of dynein is modelled by a sphere (blue) connected by two springs to the AAA+ rings. The AAA+ rings, depicted in green, and the MTBDs, depicted in yellow and orange, are modelled as spheres. The stalks are modelled as springs. The microtubule is modelled as a line (red).

for $t \in [0, T_{Final}]$ with dimensional parameter values given in Table 1 and the ranges or distributions for the stochastic parameters given in Table 2. The initial conditions can also be altered to reflect this new orientation:

$$\begin{cases} x_C(0) = 0, \\ x_T(0) = L_C, \\ x_A(0) = L_C + L_T\cos(\theta_{AT}) - 8, \\ x_B(0) = L_C + L_T\cos(\theta_{AT}), \\ x_D(0) = p_0 = L_C + L_T\cos(\theta_{AT}) + L_S\cos(\theta_{BE}) - 8, \\ x_E(0) = p_1 = L_C + L_T\cos(\theta_{AT}) + L_S\cos(\theta_{BE}). \end{cases} \tag{29}$$

The system can then be solved similarly to the original model, see Figure 6 for example profiles with $P_D = P_E = 50\%$ and Figure 7 for example profiles with $P_D = 70\%$ and $P_E = 30\%$. The results are the same as for the original model, with the percentage of not passing steps 84.57% and the percentage of alternating steps: 56.57% when $P_D = P_E = 50\%$, and the percentage of not passing steps 84.26% and the percentage of alternating steps 74.06% when $P_D = 70\%$ and $P_E = 30\%$.

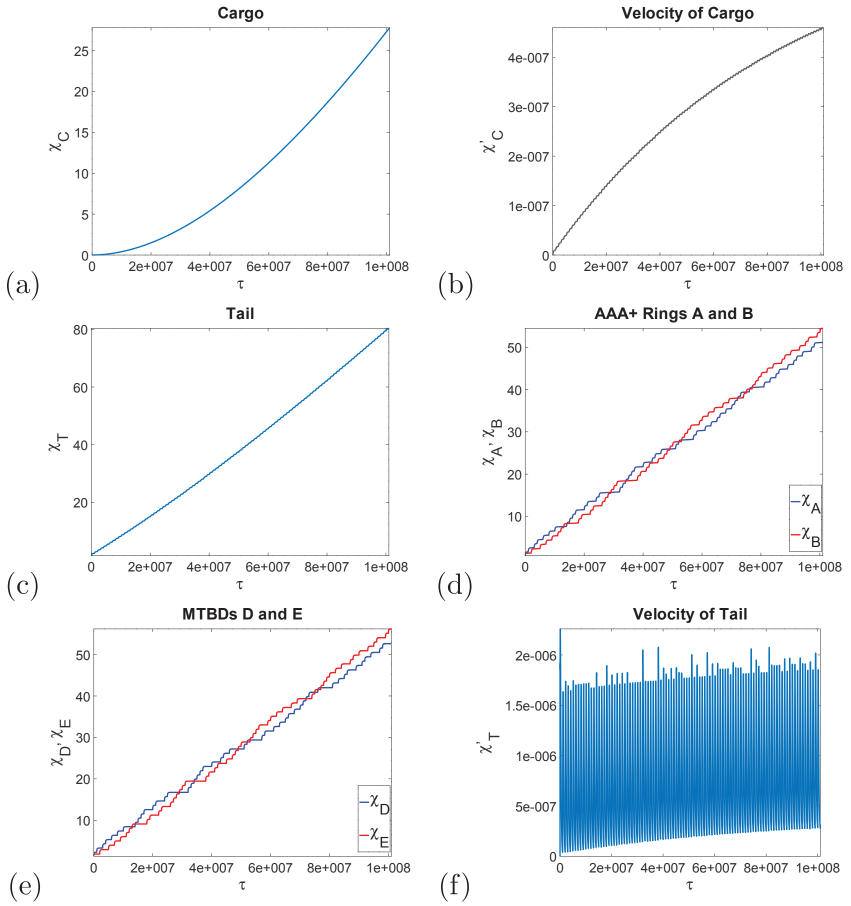

Figure 6: (Online version in colour.) Numerical solutions to the revised model with maximum separation between MTBDs at 48 nm and the probability that MTBD E steps at 50%. Plots over the whole time corresponding to (a) trajectory of the cargo, (b) velocity profile of the cargo, (c) trajectory of the tail domain, (d) trajectories of the AAA+ rings, (e) trajectories of the MTBDs, and (f) trajectory of the tail domain for a representative subinterval.

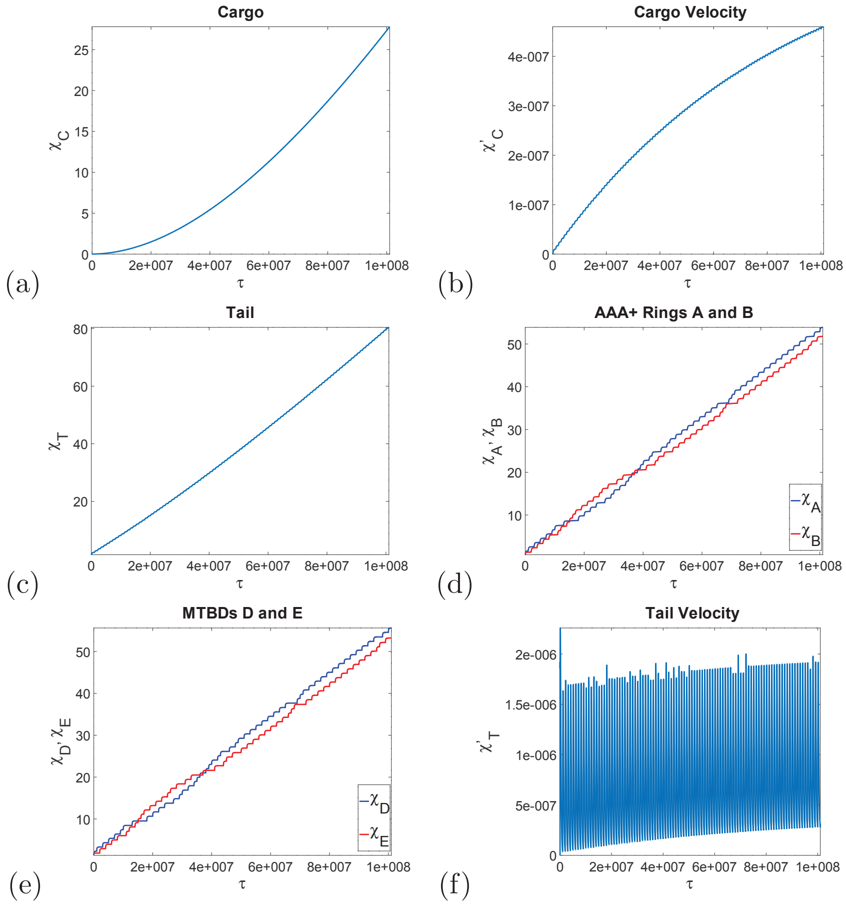

Figure 7: (Online version in colour.) Numerical solutions to the revised model with maximum separation between MTBDs at 48 nm and the probability that MTBD E steps set at 70% if the previous step was taken by MTBD D, and 30% otherwise. Plots over the whole time corresponding to (a) trajectory of the cargo, (b) velocity profile of the cargo, (c) trajectory of the tail domain, (d) trajectories of the AAA+ rings, and (e) trajectories of the MTBDs.

4 Independent Stepping

4.1 Nondimensionalization

We take a multiscale approach when nondimensionalizing the model, using one fast timescale for the stepping and one slow timescale for the dwelling. For the dwelling interval, we take $t_c = \mu$

to obtain the following system:

$$\frac{d\chi_C}{d\tau_1} = \mu\Big[\alpha_1\Big(\frac{1}{\rho_1}\chi_T - 1\Big) - \lambda_2 - \alpha_1\chi_C\Big], \tag{30}$$

$$\frac{d\chi_T}{d\tau_1} = \mu\Big[\alpha_2\Big(\frac{1}{\rho_2}(\chi_A + \chi_B) + \cos(\theta_{AT}) - \cos(\theta_{BT})\Big) + \alpha_3\rho_1(\chi_C + 1) - (2\alpha_2 + \alpha_3)\chi_T\Big], \tag{31}$$

$$\frac{d\chi_A}{d\tau_1} = \mu\Big[\alpha_4\rho_2(\chi_T - \cos(\theta_{AT})) + \alpha_5(\chi_D + \cos(\theta_{AD})) - (\alpha_4 + \alpha_5)\chi_A\Big], \tag{32}$$

$$\frac{d\chi_B}{d\tau_1} = \mu\Big[\alpha_5(\chi_E - \cos(\theta_{BE})) + \alpha_4\rho_2(\chi_T + \cos(\theta_{BT})) - (\alpha_4 + \alpha_5)\Big], \tag{33}$$

$$\frac{d\chi_D}{d\tau_1} = 0, \tag{34}$$

$$\frac{d\chi_E}{d\tau_1} = 0, \tag{35}$$

for $\tau_1 \in [\tau_{1,k}, \tau_{1,k+1}]$ where $\tau_{1,k+1} = \tau_{1,k} + \frac{1}{\mu}\min\{q_j^i : j \in \{D, E\}\}$. For the stepping intervals we take $t_c = \frac{m_C}{\gamma_C}$ and the system of ODEs is given as:

$$\alpha_C\frac{d\chi_C}{d\tau_2} = \Big(\frac{1}{\rho_1}\chi_T - 1\Big) - \lambda_1 - \chi_C, \tag{36}$$

$$\alpha_T\frac{d\chi_T}{d\tau_2} = \Big(\frac{1}{\rho_2}(\chi_B + \chi_A) - \cos(\theta_{BT}) + \cos(\theta_{AT})\Big) + \rho_1\kappa_1(\chi_C + 1) - (2 + \kappa_1)\chi_T, \tag{37}$$

$$\alpha_M\frac{d\chi_A}{d\tau_2} = \rho_2\kappa_2\Big(\chi_T - \cos(\theta_{AT})\Big) + \Big(\chi_D + \cos(\theta_{AD})\Big) - (\kappa_2 + 1)\chi_A, \tag{38}$$

$$\alpha_M\frac{d\chi_B}{d\tau_2} = \Big(\chi_E - \cos(\theta_{BE})\Big) + \rho_2\kappa_2\Big(\chi_B + \cos(\theta_{BT})\Big) - (\kappa_2 + 1)\chi_B, \tag{39}$$

$$\alpha_S\frac{d\chi_D}{d\tau_2} = h_{q,D}(\tau, \frac{\gamma_C}{m_C}\mathbf{q_D})\Big[\kappa_3(\beta_{2k} + \rho_3) + \Big(\chi_A + \cos(\theta_{AD})\Big) - (1 + \kappa_3)\chi_D\Big], \tag{40}$$

$$\alpha_S\frac{d\chi_E}{d\tau_2} = h_{q,E}(\tau, \frac{\gamma_C}{m_C}\mathbf{q_E})\Big[\kappa_3(\beta_{2k+1} + \rho_3) + \Big(\chi_B + \cos(\theta_{BE})\Big) - (1 + \kappa_3)\chi_E\Big]. \tag{41}$$

for $\tau_2 \in [\tau_{2,k}, \tau_{2,k+1}]$ where $\tau_{2,k} = \frac{\gamma_C}{m_C}\mu\tau_{1,k+1}$.

4.2 Stochastic Stepping

The initial dwell times q_D^1 and q_E^1 are generated and the system is set to dwell for $\tau \in [0, \frac{1}{\mu}\min\{q_D^1, q_E^1\}]$, hence the system is solved for the initial conditions:

$$\chi_C(0) = 0, \quad \chi_T(0) = \rho_1, \quad \chi_A(0) = \rho_2 + \rho_1\rho_2, \quad \chi_B(0) = \rho_2 + \rho_1\rho_2,$$
$$\chi_D(0) = \beta_0, \quad \chi_E(0) = \beta_1.$$

If $q_D^1 < q_E^1$ the system is then solved for MTBD D unbound for $\tau \in [\frac{\gamma_C}{m_C} q_D^1, \min\{\frac{\gamma_C}{m_C} q_E^1, \frac{\gamma_C}{m_C} q_D^1 + \tau_{step}\}]$ where we take $\tau_{Step} = 10^6$. This is necessary as the ODE system will become stiff if the timescales are too large. The initial conditions are taken to be those at the end of the dwelling interval:

$$\chi_C(\tau_{2,k}) = \chi_C(\tau_{1,k+1}), \quad \chi_T(\tau_{2,k}) = \chi_T(\tau_{1,k+1}), \quad \chi_A(\tau_{2,k}) = \chi_A(\tau_{1,k+1}),$$
$$\chi_B(\tau_{2,k}) = \chi_B(\tau_{1,k+1}), \quad \chi_D(\tau_{2,k}) = \chi_D(\tau_{1,k+1}), \quad \chi_E(\tau_{2,k}) = \chi_E(\tau_{1,k+1}).$$

for $\tau_{1,k+1} = \frac{1}{\mu}\min\{q_D^1, q_E^1\}$ and $\tau_{2,k} = \frac{\gamma_C}{m_C} q_D^1$. The solution is then truncated to the point when the MTBD reaches the binding site, time t_{bind}, a new dwell time q_D^2 is generated and we compare this dwell time with $q_E^1 - t_{bind}$. If the MTBD does not reach the binding site within the time interval, i.e. the other MTBD detaches early, then the simulation is ended. The system is solved similarly for the case $q_D^1 > q_E^1$ but with MTBD E unbound and $\tau_{2,k} = \frac{\gamma_C}{m_C} q_E^1$. This process is repeated until there have been $N = 100$ steps or both MTBDs have detached.

5 Dwelling, Backwards Stepping and Variable Step Size

Consider $t \in [0, T_F + Q_F]$ with $T_F > 0$ and $Q_F = \sum_{k=1}^{N} q_{1,k}$. Here, N represents the total number of steps, T_F the total time spent stepping and Q_F the total time spent dwelling with

$q_{1,k}$ the length of individual intervals of dwelling. Let $\mathbf{q_1} = \{q_{1,k}\}_{k=1:N}$ be a random vector where $q_{1,k}$ is from the exponential distribution with mean μ. Furthermore, let $\mathbf{q_2} = \{q_{2,k}\}_{k=1:N}$, $\mathbf{q_3} = \{q_{3,k}\}_{k=1:N}$ be random vectors where $q_{2,k}$, $q_{3,k}$ are from the uniform distribution on $(0, 1)$; these will determine the choice of AAA+ ring and the direction of stepping respectively. We continue to use a fixed time interval for stepping, T_{Step}, giving $T_F = NT_{Step}$.

For $t \in [t_i, t_{i+1}]$, where $t_{i+1} = t_i + \frac{T_F}{N}$ and given that MTBD j stepped previously, if $q_{2,k} < P_j$ then MTBD E is set to be in the unbound state and MTBD D is set to be in the bound state. Otherwise we assume that MTBD D is in the unbound state and MTBD E in the bound state. This is described by the step functions $h_{2,D}$ and $h_{2,E}$:

$$h_{2,E}(t) = \begin{cases} 1 & \text{if} \quad q_{2,k} < P_j, \\ 0 & \text{otherwise;} \end{cases} \tag{42}$$

and similarly

$$h_{2,D}(t) = 1 - h_{2,E}(t). \tag{43}$$

If the unbound MTBD is ahead of the other and the maximum separation distance has been reached or if $q_{3,k} < P_{Back}$ the unbound MTBD is set to move backwards; otherwise it steps forwards. This can be defined by the step function:

$$g_D(x_D, x_E, t, d, n) = \begin{cases} -n & \text{if} \quad x_D - x_E > d \quad \text{or} \quad q_{3,k} < P_{Back} \\ n & \text{otherwise} \end{cases} \tag{44}$$

where n is a parameter modulating the step size. The equivalent function for MTBD E can be defined similarly with

$$g_E(x_D, x_E, t, d, n) = \begin{cases} -n & \text{if} \quad x_E - x_D > d \quad \text{or} \quad q_{3,k} < P_{Back} \\ n & \text{otherwise} \end{cases} \tag{45}$$

Then, given $q_{1,k}$ the system is set to dwell for $t \in [t_{i+1}, t_{i+1} + q_{1,k}]$, i.e. both MTBDs are set to be in the bound state. Here, we can define a step function $h_q(t, t_{i+1})$ given by:

$$h_q(t, t_{i+1}) = \begin{cases} 1 & \text{if} \quad t \leq t_{i+1} \\ 0 & \text{otherwise;} \end{cases} \tag{46}$$

for $t \in [t_i, t_{i+1} + q_{1,k}]$ with $k = 1, 2, ..., N$. Define $h_{3,j}(t) = h_q(t, t_{i+1})h_{2,j}(t)$ for $j = D, E$. The system of ODEs is therefore given by:

$$m_C \frac{d^2 x_C}{dt^2} = K_C\Big(x_T - x_C - L_C\Big) - F_C - \gamma_C \frac{dx_C}{dt}, \tag{47}$$

$$\begin{aligned} m_T \frac{d^2 x_T}{dt^2} &= K_T\Big(x_B - x_T - L_T\cos(\theta_{BT})\Big) - K_T\Big(x_T - x_A - L_T\cos(\theta_{AT})\Big) \\ &\quad - K_C\Big(x_T - x_C - L_C\Big) - \gamma_T \frac{dx_T}{dt}, \end{aligned} \tag{48}$$

$$m_M \frac{d^2 x_A}{dt^2} = K_T\Big(x_T - x_A - L_T\cos(\theta_{AT})\Big) - K_S\Big(x_A - x_D - L_S\cos(\theta_{AD})\Big) - \gamma_M \frac{dx_A}{dt}, \tag{49}$$

$$m_M \frac{d^2 x_B}{dt^2} = K_S\Big(x_E - x_B - L_S\cos(\theta_{BE})\Big) - K_T\Big(x_B - x_T - L_T\cos(\theta_{BT})\Big) - \gamma_M \frac{dx_B}{dt}, \tag{50}$$

$$\begin{aligned} m_S h_{3,D}(t) \frac{d^2 x_D}{dt^2} &= h_{3,D}(t)\Big[- \gamma_{ATP} \frac{dx_D}{dt} - K_{ATP}(x_D - p_D(t) - g_D(x_D, x_E, t, d, n)L_{ATP}) \\ &\quad - K_S(x_D - x_A - L_S\cos(\theta_{AD}))\Big] - \gamma_S \frac{dx_D}{dt}, \end{aligned} \tag{51}$$

$$\begin{aligned} m_S h_{3,E}(t) \frac{d^2 x_E}{dt^2} &= h_{3,E}(t)\Big[- \gamma_{ATP} \frac{dx_E}{dt} - K_{ATP}(x_E - p_E(t) - g_E(x_D, x_E, t, d, n)L_{ATP}) \\ &\quad - K_S(x_E - x_B - L_S\cos(\theta_{BE}))\Big] - \gamma_S \frac{dx_E}{dt}, \end{aligned} \tag{52}$$

for $t \in [0, T_F + Q_F]$. Here, $p_D(t)$ and $p_E(t)$ represent the binding sites for MTBDs D and E respectively, where $p_D(0) = p_0$, $p_E(0) = p_1$ and the subsequent choice of binding site will depend on the previous step size and direction of the respective MTBD; i.e. if MTBD D steps at time t_i with a step of $\pm nL_{ATP}$ then the new binding site is taken to be $p_D(t_{i+1}) = p_D(t_i) \pm nL_{ATP}$. See Table 1 in the main paper for dimensional parameter values and Table 2 for the ranges and distributions of stochastic parameters. The system is then nondimensionalized similarly to Section 4.1 above.

5.1 Stochastic Stepping

For the initial step MTBD D is assumed to be in the unbound state and MTBD E is in an bound state and the system is solved for the initial conditions:

$$\chi_C(0) = 0, \quad \chi_T(0) = \rho_1, \quad \chi_A(0) = \rho_2 + \rho_1\rho_2, \quad \chi_B(0) = \rho_2 + \rho_1\rho_2,$$
$$\chi_D(0) = \beta_0, \quad \chi_E(0) = \beta_1.$$

For each subsequent step a random number $q_{1,k}$ is generated from the exponential distribution with mean μ and the system of ODEs is solved for $\tau \in [\tau_{i+1}, \tau_{i+2}]$ in the dwelling state where $\tau_{i+2} = \tau_{i+1} + \frac{1}{\mu}q_{1,k}$. Initial conditions are given by the values from the previous simulation:

$$\chi_C(\tau_{i+1}), \quad \chi_T(\tau_{i+1}), \quad \chi_A(\tau_{i+1}), \quad \chi_B(\tau_{i+1}), \quad \chi_D(\tau_{i+1}) \quad \text{and} \quad \chi_E(\tau_{i+1}).$$

Random numbers $q_{2,k}$, $q_{3,k}$ are then generated from the uniform distribution on $(0, 1)$ to determine which head domain steps and in which direction. We take n from a Poisson distribution about 2 for the forward steps and from the Poisson distribution about 1 for the backward steps. The resulting ODE system is solved for $\tau \in [\tau_{i+2}, \tau_{i+3}]$, where $\tau_{i+3} = \tau_{i+2} + \tau_{step}$, with the initial conditions taken from the end values of the dwelling simulation:

$$\chi_C(\tau_{i+2}), \quad \chi_T(\tau_{i+2}), \quad \chi_A(\tau_{i+2}), \quad \chi_B(\tau_{i+2}), \quad \chi_D(\tau_{i+2}) \quad \text{and} \quad \chi_E(\tau_{i+2}).$$

6 MTBDs vs Rings

The behaviour of the MTBDs and the AAA+ rings demonstrate some differences with regards to not passing steps, however these only become significant for small values of the maximum separation distance (see Table 1) and do not depend on the stepping probabilities (see Table 2); there is no difference for alternating steps. For the model with large scale dwelling, variable step sizes and backwards stepping, there is a 4% difference in not passing steps with 82.3% for the MTBDs and 86.8% for the AAA+ Rings.

Table 1: Mean percentage of not passing steps given a range of values for the maximum separation distance d (nm). The data represents the results of 100 simulations with the probability that MTBD E steps set at 74% if MTBD D stepped previously and 26% otherwise. The probability of random backwards stepping is set to be 10% and the mean dwell time is taken to be 2 ns. If x% of steps are not passing then $(100 - x)$% of steps are passing. Similarly, if x% of steps are alternating then $(100 - x)$% of steps are not alternating.

d (nm)	MTBDs (%)	AAA+ Rings(%)
8	49.36	95.43
16	53.57	81.25
24	68.05	79.35
32	75.45	76.32
40	80.85	84.15
48	81.68	83.86
56	84.50	89.50
64	85.72	87.62
72	85.89	89.57
80	87.29	89.00

Table 2: Mean percentage of not passing steps given a range of values for the stepping probabilities of MTBD E. The data represents the results of 100 simulations with the maximum separation distance set to be 56 nm. The probability of random backwards stepping is set to be 10% and the mean dwell time is taken to be 2 ns. Observe that the % of not passing steps is independent of the probabilities P_D and P_E while the % of alternating steps is closely related. If x% of steps are not passing then $(100-x)$% of steps are passing. Similarly, if x% of steps are alternating then $(100-x)$% of steps are not alternating.

P_D (%)	P_E (%)	MTBDs	AAA+ Rings
20	80	88.00	89.02
30	70	87.13	88.82
40	60	87.29	89.53
50	50	86.23	87.02
60	40	85.87	87.26
70	30	84.17	86.44
80	20	84.81	86.84
